# Effect of a Simple Information Booklet on Pain Persistence after an Acute Episode of Low Back Pain: A Non-Randomized Trial in a Primary Care Setting

**DOI:** 10.1371/journal.pone.0000706

**Published:** 2007-08-08

**Authors:** Emmanuel Coudeyre, Florence Tubach, François Rannou, Gabriel Baron, Fernand Coriat, Sylvie Brin, Michel Revel, Serge Poiraudeau

**Affiliations:** 1 Centre de Médecine Physique et Réadaptation Notre Dame, Chamalières, France; 2 Service de Médecine Physique et Réadaptation, Hôpital Cochin AP-HP, Université Paris 5, Institut Fédératif de Recherche Handicap (IFR 25) INSERM, Paris, France; 3 INSERM U738, Département d'épidémiologie, biostatistique et recherche clinique, Hôpital Bichat, Paris, France; 4 Laboratoire Aventis, Groupe sanofi-aventis, Paris, France; Institut Universitaire de Médecine Sociale et Préventive, Switzerland

## Abstract

**Objective:**

Mass-media campaigns have been known to modify the outcome of low back pain (LBP). We assessed the impact on outcome of standardized written information on LBP given to patients with acute LBP.

**Methods:**

*Design:* A 3-month pragmatic, multicenter controlled trial with geographic stratification. *Setting:* Primary care practice in France. *Participants:* 2752 patients with acute LBP. *Intervention:* An advice book on LBP (the “back book”). *Main outcome measures:* The main outcome measure was persistence of LBP three months after baseline evaluation.

**Results:**

2337 (85%) patients were assessed at follow-up and 12.4% of participants reported persistent LBP. The absolute risk reduction of reporting persistent back pain in the intervention group was 3.6% lower than in the control group (10.5% vs. 14.1%; 95% confidence interval [−6.3% ; −1.0%]; p value adjusted for cluster effect = 0.01). Patients in the intervention group were more satisfied than those in the control group with the information they received about physical activities, when to consult their physician, and how to prevent a new episode of LBP. However, the number of patients who had taken sick leave was similar, as was the mean sick-leave duration, in both arms, and, among patients with persistent pain at follow-up, the intervention and control groups did not differ in disability or fear-avoidance beliefs.

**Conclusions:**

The level of improvement of an information booklet is modest, but the cost and complexity of the intervention is minimal. Therefore, the implications and generalizability of this intervention are substantial.

**Trial Registration:**

ClinicalTrials.gov NCT00343057

## Introduction

About 60% of the population is concerned with low back pain (LBP) in Western industrialized countries [1]. Chronic LBP (cLBP) has become a major medical, social and economic problem [2]. The costs are comparable to those induced by coronary heart disease, diabetes, or depression [3]. Diminishing the cost is a major public health issue. An approach to achieving this goal is to improve the prevention of chronic disease in patients with acute LBP in primary care practice.

Psychosocial factors have been shown to be associated with the development of disability with cLBP [4], and the best individualized factors are anxiety, depression, coping, and fear of and belief about pain [5]. Providing advice to stay active and information about how to cope with pain has been shown to modify patients' fears, avoidance attitudes and beliefs [6], [7]. Because health care providers play a pivotal role in patient information and education in primary care practice, general practitioners (GPs) could greatly influence the outcome in LBP and thus contribute to decreased LBP-related costs.

Several guidelines and reviews on the management of acute LBP and cLBP in primary care practice are available [8]–[25]. They mainly concern prescriptions for X-rays and laboratory tests, treatment options, and information about physical and occupational activities. The consensus among international guidelines seems to be that advice to stay active, reassurance and use of analgesics, if necessary, for pain relief are important for acute episodes of LBP. Specific exercises and occupational activities seem to be useful for persistent but not acute LBP. Exercises, education about back pain, behavioural treatment and multidisciplinary treatment seem effective in preventing persistent/chronic LBP. Concerning information given to patients, evidence suggests that advice about staying active and coping with pain could decrease the rate of patients experiencing chronic pain and the impact of LBP on daily and occupational activities [23]–[25].

Information booklets, the lowest-cost information tools, have been developed to help health care providers inform and educate patients. Among these booklets [7], [23]–[25], the “back book,” developed from evidence-based medicine by a multidisciplinary team and published to accompany the United Kingdom back-pain guidelines [26], has been shown, in a limited sample of English patients with LBP, to significantly affect fears and beliefs about and disability with LPB but not pain level [7]. Other educational booklets or pamphlets have been previously assessed, with relatively modest effects, for acute [27], chronic [28], or occupational [29] back pain. The “back book” has been translated with use of a validated translation/back translation procedure and culturally adapted to French-speaking patients with LBP [30]. It is the only evidence-based information booklet available for such patients (i.e., “le Guide du dos”) but has never been evaluated in this setting.

The aim of this study was to assess the impact of the “back book” on outcome (persistence of pain at 3 months) in acute LBP in a French national sample of patients in a primary care setting.

## Methods

The protocol for this trial and supporting CONSORT checklist are available as supporting information; see Checklist S1 and Protocol S1.

### Trial design

We conducted a 3-month prospective, controlled study with a quasi-experimental design (i.e., a nonrandomized controlled sample with geographic stratification [30 areas]). Control and intervention areas were selected for their similarities in rural-to-urban distribution of the population and patients' access to GPs and to minimize risk of overlap between areas.

### Participants

#### GPs

A total of 60 GPs per geographic area were selected at random from a national database (Logimed) according to a computerized allocation [31], and 1800 GPs were invited to participate in the study. Those agreeing to participate were assigned to the intervention or control group, depending on their geographic area. Each GP in the intervention group received a personal copy of the “back book” and was asked to read it before including patients and explaining the booklet to patients. GPs in the control group were told that they would get the book at the end of the trial.

#### Patients

Each GP had to enrol up to 4 consecutive patients with acute LBP. Patients were excluded if they (a) were less than 18 years old; (b) had pain for more than 4 weeks; (c) had pain intensity for the previous 24 hours less than 3 on a 11-point numeric scale (0 = no pain, 10 = maximal pain); (d) had sciatica; (e) had had a previous episode of acute LBP during the last 12 months; (f)did not work; (h) had consulted another practitioner for the same episode of back pain; (i) were pregnant; (j) had back pain related to infection, tumor, or inflammatory disease; or (k) had previously undergone back surgery.

### Ethical approval

The study protocol was approved by the Commission Nationale Informatique et Liberté and the French National Medical Council (Conseil National de l'Ordre des Médecins). The study was conducted in compliance with the protocol Good Clinical Practices and Declaration of Helsinki principles. In accordance with the French national law, GPs and patients gave their written consent to participate after being informed about the study protocol.

### Intervention

At baseline, all patients received medical care and oral information as usually provided by GPs. In the intervention group, all patients also received the “back book.”

### Outcome measures

#### Main outcome measure

To assess persistence of LBP 3 months after baseline evaluation, patients were asked to answer Yes or No to the question “Has your low back pain persisted since the first visit to your GP 3 months ago?” Patients answering Yes were considered as having persistent back pain.

#### Physician questionnaire

Parts 1, 2, and 3 of the physician self-administered questionnaire completed at baseline concerned demographic data (age and sex), professional data (years of practice and exclusively private or public/private practice) and personal history of LBP (acute, recurrent, chronic), and self-limitation of physical activities for LBP (never, sometimes, often, always), respectively. Part 4 dealt with GPs' formation of practice and practice for LBP: participation in an educational session on LBP in the last 3 years (yes/no); mean length of sick leave prescription for acute LBP if needed (≤3 days, >3 and ≤8 days, >8 days), advice about physical activities during sick leave for acute LBP (bed rest, rest at home, keep maximum bearable activities). Part 5 assessed GPs' fears, avoidance attitudes and beliefs on the Fear-Avoidance Beliefs Questionnaire (FABQ)[5], which consists of 2 independent subscales: FABQ Phys and FABQ Work. The FABQ Phys assesses fears, avoidance attitudes and beliefs related to general physical activities (4 items, range 0–24), and the FABQ Work assesses fears, avoidance attitudes and beliefs related to occupational activities (7 items, range 0–42). Each item is scored from 0, “do not agree at all,” to 6, “completely agree”. For both subscales, a low score indicates low fears, avoidance attitudes and beliefs. According to the designers of the scale, a score of 14 or more on the FABQ Phys scale indicates strong beliefs [5], [7]. This questionnaire has been validated in English [5], German [32], and, recently, in French [33]. The FABQ was originally developed to assess patients' fears, avoidance attitudes and beliefs. To evaluate GPs' fears, avoidance attitudes and beliefs, we did not modify the phrasing of items but slightly adapted the first sentence of instructions to patients. This sentence was “these are statements that other patients have expressed about their low back pain…”; we just deleted the word “other”.

#### Patients' questionnaire

##### Baseline evaluation

Baseline data were collected during the first visit to GPs. Patients were interviewed about pain intensity (11-point numeric scale, from 0, no pain, to 10, maximal pain), physical demand of occupational activities (11-point numeric scale, from 0, no physical demand, to 10, extremely hard physical demand), education level (no full-time education, primary school, high school, post-graduate education), presence of LBP in parents (yes/no), length of back pain (days), work-related back pain (yes/no), sport activities (none, occasional, regular, competition), medication intake for the previous week (analgesics, nonsteroidal anti-inflammatory drugs [NSAIDs], muscle relaxants), pain intensity for the last 48 hours (weak, moderate, severe, extremely severe), and handicap level for activities of daily living (no handicap, weak, moderate, severe, extremely severe). Self-rated disability was assessed on the Quebec questionnaire (20 items scored from 0, no disability, to 5, impossible to do; range 0–100) [34]. LBP beliefs were recorded on the FABQ (see GPs' questionnaire).

##### 3-month follow-up evaluation

At baseline, an appointment for a follow-up visit with the GP was established for the patient. Follow-up data were recorded during this visit to the GP (77% of patients) or by phone interviews (23% of patients), conducted by trained research assistants for patients who did not consult their physician. Patients were asked about persistence of LBP since the baseline evaluation (yes/no); whether radiography, computed tomography (CT), or magnetic resonance imaging had been performed (yes/no); sick leave since baseline evaluation (yes/no); sick leave duration (days); return to work (yes/no); and satisfaction with information received about physical activities, when to see a doctor, how to use medications, and how to prevent new episodes of LBP (very satisfied, rather satisfied, rather not satisfied, unsatisfied). For patients with persistent LBP, pain intensity, level of handicap, perceived disability, anxiety and depression, and LBP beliefs were recorded as during the baseline evaluation.

### Sample size

Persistent LBP after an acute episode has previously been reported to occur in 7% of patients in France [35]. We hypothesized (consensus among the authors) that a difference of 3 points between control and intervention groups (7% in the control and 4% in the intervention groups) would have a clinical and public health relevance. With a 2-sided chi-square test at 5%, a sample size of 1212 patients per group would have 90% power for detecting a significant difference between the control and intervention groups. Allowing for 15% of patients lost to follow-up at 3 months, we sought to recruit 2830 patients.

### Statistical analysis

The impact of the “back book” intervention was assessed by comparing the proportions of patients with persistent LBP after 3 months who had been exposed to the “back book” and those who had not been exposed. Qualitative outcomes were compared by chi-square test and quantitative outcomes by *t*-test. To take into account the cluster effect (patients are clustered within physician), the primary outcome (persistent pain) was also analysed in the framework of a generalized estimation equation (GEE) regression model, with the physician considered as a random effect. The same procedure was applied to assess the impact of the “back book” on the secondary outcomes.

The analysis was per protocol. We performed 2 intention-to-treat analyses. In the first analysis, we considered all patients lost to follow-up as having persistent pain at 3 months. In the second, we considered all patients lost to follow-up as having no persistent pain at 3 months.

Data analyses involved use of SAS (SAS institute Inc, Cary, NC).

## Results

### Flow of participants through trial

**Figure 1:**
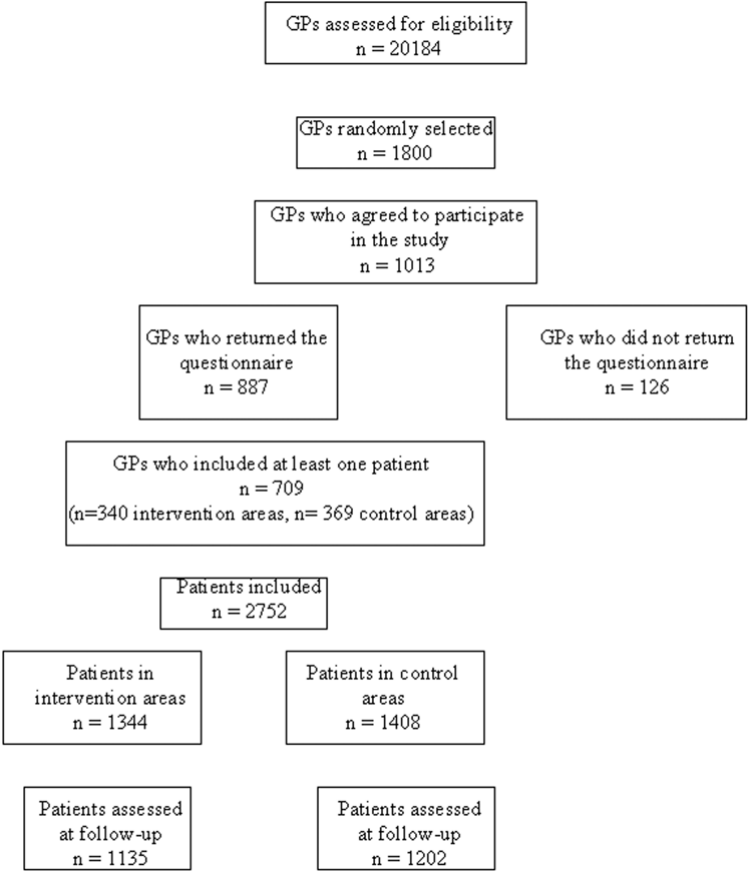
Flow of general practitioners and patients through the trial.

The Logimed database contains information on 20 184 GPs. A total of 60 GPs per geographic area were selected at random from the database and asked to participate, with 1013 GPs agreeing to participate. Of these, 88% returned their questionnaires, and 709 (70%) included at least 1 patient (Figure 1). Among the GPs who agreed to participate, 488 were in intervention areas and 525 in control areas, and 69.7% of GPs in the intervention and 70.3% in the control areas returned their questionnaires and included at least one patient.

A total of 2752 patients met the inclusion criteria, and 2337 (82.6%) (1135 and 1202 in the intervention and control groups, respectively) were assessed at follow-up. At baseline, patients lost to follow-up did not differ from those assessed at 3 months in terms of age, gender, pain and handicap levels, sport activities, education level, disability, and beliefs (table 1). The number of patients lost to follow-up did not differ between the intervention and control groups (209 [15.5%] and 206 [14.6%] respectively).

**Table 1:** Baseline demographic and clinical characteristics of patients

	Patients assessed at follow-up N = 2337	Intervention group N = 1135	Control Group N = 1202	Lost to follow-up N = 412
**Age** (m±SD), years	45±12	45±12	44±12	43±12
**Sex** (M)	1321 (57%)	628 (56%)	693 (58%)	237 (57%)
**Back pain duration** (median (interquartile range), **days**	3 (5)	3 (4)	4 (5)	3 (6)
**Physical demand of work (0–10)** (m±SD)	5.7±2.5	5.7±2.5	5.7±2.5	5.5±2.6
**Education level**				
No full-time education	51 (2%)	27 (2%)	24 (2%)	7 (2%)
Primary school	656 (28%)	294 (26%)	362 (30%)	114 (28%)
High school	1075 (46%)	528 (47%)	547 (46%)	207 (50%)
Post graduate	552 (24%)	284 (25%)	268 (22%)	86 (20%)
**Back pain in parents**	1242 (54%)	593 (53%)	649 (55%)	198 (49%)
**Work-related back pain**	193 (8%)	95 (8%)	98 (8%)	37 (9%)
**Sports activities**				
None	1061 (45%)	502 (45%)	559 (47%)	199 (48%)
Occasional	830 (36%)	411 (36%)	419 (35%)	133 (32%)
Usual	414 (18%)	207 (18%)	207 (17%)	76 (18%)
Competition	21 (1%)	10 (1%)	11 (1%)	4 (2%)
**Medications** *				
Analgesics (yes)	1903 (81%)	929 (82%)	974 (81%)	322 (78%)
NSAIDs (yes)	611 (26%)	286 (25%)	325 (27%)	102 (25%)
Muscle-relaxants (yes)	434 (19%)	196 (17%)	238 (20%)	87 (21%)
**Pain level (m±SD)**	6.8±1.5	6.7±1.5	6.8±1.5	6.7±1.5
**Pain intensity**				
None/weak	49 (2%)	24 (2%)	25 (2%)	11 (5%)
Moderate	485 (21%)	229 (20%)	256 (21%)	85 (21%)
Severe	1598 (69%)	777 (70%)	821 (69%)	276 (68%)
Extremely severe	181 (8%)	90 (8%)	91 (8%)	24 (6%)
**Handicap level** **				
None/weak	78 (3%)	35 (3%)	43 (4%)	14 (3%)
Moderate	704 (30%)	328 (28%)	376 (31%)	131 (32%)
Severe	1352 (59%)	671 (60%)	681 (57%)	236 (59%)
Extremely severe	178 (8%)	85 (8%)	93 (8%)	23 (6%)
**LBP beliefs**				
FABQ Phys (0–24) (m±SD)	16.9±5.0	16.7±5.0	17.1±4.9	16.2±5.0
FABQ Work (0–42) (m±SD)	19.7±10.9	19.7±11.1	19.7±10.6	18.8±10.8
**Disability**				
Quebec (0–100) (m±SD)	55.0±17.2	55.3±16.9	54.7±17.6	54.6±17.6

Values are number of patients (percentages)

* for the last week

** for activities of daily living

NSAID = nonsteroidal anti-inflammatory drug; FABQ = Fear-Avoidance Beliefs Questionnaire

### Baseline characteristics

Patients (Table 1) and GPs (Table 2) in the intervention and control groups did not differ in baseline characteristics. The patients' mean (±SD) age was 45±12 years, and 57% were male. The median pain duration at baseline was 3 (median interquartile range 5) days and pain level 6.8±1.5 (range 0–10). Before seeing their GP, 81% of patients had taken analgesics, 26% NSAIDs, and 19% muscle relaxants. The Quebec disability scale mean score was 55.0±17.2 (range 0–100). Fears and beliefs were high, with FABQ Phys and Work mean scores of 16.9±5.0 and 19.7±10.9, respectively. A total of 72% of patients had strong fears and beliefs about back pain [5], [7]. The GPs' mean age was 48±7 years; 79% in both groups were male, 69% worked in an urban environment, and 87% had more than 10 years of practice.

**Table 2:** Demographic and professional characteristics, and personal history of back pain of GPs, GPs' formation of and self-reported attitude about acute low back pain, and GPs' self-reported recommendations for chronic low back pain

	Whole sample of GPs N = 709	Intervention practices N = 340	Control practices N = 369	p-value (t-test or Chi2)
**Age** mean (SD)	48.0 (7.0)	48.2 (6.6)	47.9 (7.4)	p = 0.71
**Gender** (M)	563 (79.5%)	269 (79.1%)	294 (79.9%)	p = 0.80
**Years of practice**				
<10	93 (13.2%)	39 (11.6%)	54 (14.7%)	p = 0.16
10–20	296 (42.0%)	155 (46.0%)	141 (38.4%)	
21–30	279 (39.6%)	129 (38.3%)	150 (40.9%)	
>30	36 (5.2%)	14 (4.1%)	22 (6.0%)	
**Environment of practice**				
Rural	216 (30.5%)	116 (34.1%)	100 (27.1%)	p = 0.10
Urban	390 (69.1%)	222 (65.3%)	268 (72.6%)	
Rural and urban	3 (0.4%)	2 (0.6%)	1 (0.3%)	
**Personal history of back pain**				
Acute	353 (49.9%)	171 (50.4%)	182 (49.3%)	p = 0.77
Recurrent	234 (33.0%)	108 (31.9%)	126 (34.2%)	p = 0.52
Chronic	102 (14.4%)	52 (15.3%)	50 (13.6%)	p = 0.49
**Self-limitation of physical activity for back pain**				
Never	215 (49.9%)	96 (47.5%)	119 (52.0%)	p = 0.61
Rarely	185178 (42.9%)	90 (44.6%)	95 (41.5%)	
Frequently	23 (5.3%)	13 (6.4%)	10 (4.4%)	
Always	8 (1.9%)	3 (1.5%)	5 (2.1%)	
**GPs**' **education about back pain**				
Education session on back pain in the last 3 years (yes)	316 (46.4%)	154 (47.2%)	162 (45.6%)	p = 0.67
**GPs**' **attitude about back pain**				
Specific information delivered (yes)	586 (85.8%)	281 (85.9%)	305 (85.7%)	p = 0.92
Recommended sick leave duration for acute back pain				
≤3 days	59 (8.4%)	30 (9.0%)	29 (7.9%)	p = 0.54
3–8 days	570 (81.3%)	266 (79.6%)	304 (82.8%)	
>8 days	72 (10.3%)	38 (11.4%)	34 (9.3%)	
Physical activities recommended during sick leave for acute back pain				
Bed rest	37 (5.4%)	17 (5.2%)	20 (5.5%)	P = 0.13
Rest	454 (65.7%)	204 (62.2%)	250 (68.9%)	
Maximum bearable activity	200 (28.9%)	107 (32.6%)	93 (25.6%)	
** GPs**' **FABQ Phys score (range 0–24)**	9.6 (4.8)	9.2 (4.5)	10.0 (5.0)	P = 0.10
** GPs' FABQ Work score (range 0–42)**	17.5 (6.7)	17.6 (6.7)	17.4 (6.6)	P = 0.69

Values are numbers (percentages) or m±SD

### Impact of the intervention at 3-month follow-up

At 3-month follow-up, 12.4% of participants reported persistent LBP. In the intervention group, the proportion of patients experiencing persistent back pain was lower than in the control group (10.5% vs. 14.1%; difference −3.6%; 95% CI [−6.3% ; −1.0%]) (Table 3). The outcome was different depending on the method used to collect data for the primary outcome. In the intervention group, among patients reporting persistent pain, only 7.6% were assessed by phone interviews as compared with 25.6% of those free of pain. A similar proportion was observed in the control group, with 13.6% and 24.2% for those reporting pain and those free of pain, respectively. In the intervention group, persistent pain was reported by 3.5% of patients assessed by phone and 12.7% by face-to-face interview. In the control group, persistent pain was reported by 8.4% of those assessed by phone and 15.7% by face-to-face interview. Finally, the method of collecting data did not differ between groups (23.7% and 22.7% in the intervention and control groups were assessed by phone interview, respectively). For the current LBP episode, patients in the intervention group were not less often referred to a spine specialist and had a similar rate of spine imaging exams as patients in the control group. The number of patients who had taken sick leave was similar, as was the mean sick-leave duration in both arms. Fewer patients had taken NSAIDs and muscle relaxants in the intervention group than the control group. Patients in the intervention group were more satisfied than those in the control group about the information and advice they received about physical activities, when to consult their physician, and how to prevent a new episode of LBP but not more satisfied about advice on medication intake. Adjusting for the cluster effect did not change the crude results, except for referring to a spine specialist.

**Table 3:** Follow-up assessment at 3 months

	Whole sample N = 2337	Intervention group N = 1135	Control group N = 1202	Percentage or mean difference (95% CI)	P value* (Chi-square or t-test)	P value** (adjusted for cluster effect)
Persistent back pain	289 (12.4%)	119 (10.5%)	170 (14.1%)	−3.6 (−6.3; −1.0)	0.0072	0.0131
Visit to spine specialist	680 (29.4%)	305 (27.2%)	375 (31.5%)	−4.3 (−8.0 ; −0.5)	0.0253	0.0566
Spine imaging						
Plain radiography	676 (29.2%)	327 (29.1%)	349 (29.2%)	−0.1 (−3.8 ; 3.6)	0.9428	0.8099
Computed tomography	194 (8.4%)	89 (7.9%)	105 (8.8%)	−0.9 (−3.1 ; 1.4)	0.4469	0.4379
Magnetic resonance imaging	45 (1.9%)	24 (2.1%)	21 (1.8%)	0.3 (−0.7 ; 1.5)	0.5114	0.6886
Sick leave						
Yes	1037 (44.9%)	503 (45.0%)	534 (44.8%)	0.2 (−4.9 ; 4.3)	0.9250	0.9132
Sick leave duration (m±SD)	6.5±14.4	6.0±12.9	6.9±15.7	−0.9 (−2.0 ; 0.4)	0.1592	0.2847
**Analgesic intake**	1964 (84.0%)	936 (82.5%)	1028 (85.5%)	−3.0 (−6.0 ; −0.1)	0.0437	
**NSAID intake**	1008 (43.1%)	447 (39.4%)	561 (46.7%)	−7.3 (−11.3 ; −3.3)	0.0004	0.0103
**Muscle-relaxant intake**	864 (37.0%)	374 (32.9%)	490 (40.8%)	−7.9 (−11.7 ; −3.9)	<0.0001	0.0176
**Information about Physical activities**					0.0003	0.0020
Very satisfied	1699 (73.7%)	868 (77.4%)	831 (70.3%)	7.1 (3.5 ; 10.7)		
Rather satisfied	437 (19.0%)	194 (17.3%)	243 (20.5%)	−3.6 (−6.4 ; −0.1)		
Rather unsatisfied	152 (6.6%)	55 (4.9%)	97 (8.2%)	−3.2 (−5.3 ; −1.3)		
Unsatisfied	17 (0.7%)	5 (0.4%)	12 (1.0%)	−0.6 (−1.3 ; 0.1)		
**When to consult a physician**					0.0010	0.0052
Very satisfied	1428 (62.0%)	739 (66.0%)	689 (58.2%)	7.8 (3.8 ; 11.8)		
Rather satisfied	632 (27.5%)	281 (25.1%)	351 (29.7%)	−4.6 (−8.2 ; −1.0)		
Rather unsatisfied	215 (9.3%)	86 (7.7%)	129 (10.9%)	−3.2 (−5.6 ; −0.8)		
Unsatisfied	27 (1.2%)	13 (1.3%)	14 (1.2%)	0.1 (−0.9 ; 0.9 )		
**How to prevent a new episode of back pain**					0.0033	0.0243
Very satisfied	1290 (56.0%)	660 (58.8%)	630 (53.3%)	5.5 (1.5 ; 9.6)		
Rather satisfied	750 (32.6%)	359 (32.0%)	391 (33.1%)	−1.1 (−4.9 ; 2.7)		
Rather unsatisfied	245 (10.6%)	97 (8.7%)	148 (12.5%)	−3.8 (−6.4 ; −1.4)		
Unsatisfied	19 (0.8%)	6 (0.5%)	13 (1.1%)	−0.6 (−1.3 ; 0.2)		
**Medication intake**					0.7090	0.9901
Very satisfied	1531 (66.4%)	743 (66.3%)	788 (66.6%)	−0.3 (−4.2 ; 3.5)		
Rather satisfied	622 (27.0%)	309 (27.5%)	313 (26.5%)	1.0 (−2.5 ; 4.7)		
Rather unsatisfied	142 (6.2%)	66 (5.9%)	76 (6.4%)	−0.5 (−2.5 ; 1.4)		
Unsatisfied	9 (0.4%)	3 (0.3%)	6 (0.5%)	−0.2 (−0.8 ; 0.3)		

Values are numbers (%); NSAID = nonsteroidal anti-inflammatory drug

* P value when computing absolute difference

** P value when taking into account the cluster effect

For intention-to-treat analysis on the main outcome measure, we used sensitivity analysis on the primary outcome by considering two extreme scenarios (worst and best-case imputation, respectively). In the first scenario, we considered that all patients lost to follow-up had persistent back pain at 3 months. The proportion of patients reporting persistent back pain at 3 months was lower in the intervention than in the control group, but the difference was not significant (24.4% versus 26.7%; difference −2.3%; 95% CI [−1.0 ; 5.6]). However, for this scenario, the power of the test to detect a statistically significant difference was only 25%. In the second scenario, we considered that all patients lost to follow-up had no persistent back pain at 3 months. The proportion of patients reporting persistent back pain was lower in the intervention than in the control group, but the difference was significant (8.9% versus 12.1%; difference −3.2%; 95% CI [−5.5 ; −1.0]). For this scenario, the power of the test was 70%.

Among patients with persistent pain at follow-up, the intervention and control groups did not differ in disability (Quebec mean score 31.0±14.7 and 31.3±15.5, respectively) or fear-avoidance beliefs (FABQ Phys and Work mean scores 12.0±5.3 and 13.3±5.6, and 19.1±10.3 and 19.8±11.6, respectively).

## Discussion

### Interpretation

Our results suggest that providing the “back book” to patients consulting their GPs for acute LBP can reduce the proportion of patients reporting persistent pain at 3 months by 3.6% as compared with patients who did not receive the book. Previous studies have demonstrated a positive impact of such books for patients [7] or physicians [36] in terms of beliefs about LBP, patients' satisfaction with information [23], [24], patients' knowledge about LBP [25], and number of visits to GPs [37]. However, we failed to demonstrate any effect of the book on disability and pain level in patients reporting persistent pain and, as others, on work absenteeism [29]. Our study is the first to report the impact of this simple educational intervention on the outcome of patients with acute LBP in a large national sample. The “back book” has also been used as a part of an information intervention including radio and TV spots and newspaper advertising in a large mass-media campaign about LBP in Australia, which had a short- [6] and long-term positive impact [38] on claims and costs related to LBP. These multimedia interventions are difficult to perform and very expensive, but booklets or pamphlets are simple to use and inexpensive.

The “back book” may have several other positive effects. We observed a decrease of NSAID and myo-relaxant but not analgesic intake in the intervention group. This observation is not surprising, since advice about pain medications in this booklet recommends grade I analgesics, especially paracetamol. The other positive effect was patients' satisfaction with information. Most of patients' complaints and claims about medical procedures relate to lack of information [39].

However, overall the “back book” had no effect on the other main back-pain outcomes: the number of sick leaves taken or sick-leave duration and perceived disability in patients reporting persistent pain. This observation had already been observed after occupational low back injury [29]. More complex and expensive interventions involving several participants may be required to reach this objective. As compared with actual experience and behavioural experiments, education seems to be a weak intervention for changing attitudes and beliefs with cLBP [40]. Despite the existence of guidelines [8], the mean sick-leave duration reported in this study may seem quite long. This finding may be explained, at least in part, by the French sick-leave policy: GPs are free to order sick leave and, until recently, sick leaves of less than one month received almost no monitoring. Furthermore, during the first 3 months of sick leave, most employees receive their entire salary.

The “back book” also had no effect on the rate of prescriptions to undergo spine imaging. Almost 29% of patients underwent plain radiography and 8% CT. Thus, GPs are not following guidelines [8], [9], which emphasizes the need for large-scale education programs on this topic.

Finally, the effects of the “back book” may be underestimated in this study, since GPs in the control group knew that the trial assessed the impact of information on patients with LBP and may have followed the guidelines more closely than under real-life conditions.

Another important result is the high proportion of patients reporting persistent pain after 3 months in the control group. This proportion probably differs among countries [41], and the only information available for France at the beginning of our study was 6.2% of patients consulting their GP for an acute episode of LBP having persistent LBP 7 weeks later [35]. The difference between our results and those from the previous French study may seem paradoxical, since some of the patients in the previous study had severe symptoms (sciatica). The principal explanation for the difference probably lies in the definition of persistent back pain between the 2 studies: no pain in our study (a stricter definition, as patients undoubtedly define it), and no decrease in pain in the previous study. Finally, in the first French study, only 64% of patients were totally free from LBP.

### Limitations

Randomized controlled trials are widely accepted as the criterion standard in assessing the effectiveness of specific therapies [42]. However, nonrandomized evaluation design is now widely accepted as quasi-experimental design that can contribute important data on the efficacy or effectiveness of interventions [43], [44], especially to evaluate public health interventions [31]. Moreover, quasi-experimental study designs that use control groups and pretests are considered to be the soundest of nonrandomized evaluations in terms of establishing causality [45]. In our study, the individual randomization of patients was not appropriate, since the GP was considered part of the intervention, which mainly consisting of information and education. GPs in the intervention arm were given the “back book” and those in the control group were not. Furthermore, during the course of our study, no LBP-centred intervention (press release or Heath Care Services intervention) that could have affected the evaluation of the impact of the “back book” was implemented in any of the included geographic areas. Finally, we did not perform a cluster randomized study by randomizing GPs practices to intervention or control because we assumed that the risk of contact between GPs in control and intervention practices during the trial would have been higher than with a quasi-experimental design with geographic stratification.

The main analysis was conducted on a per protocol population. However, the proportion of patients lost to follow-up (15%) is acceptable and was similar in both arms. Moreover, patients lost to follow-up did not differ from those evaluated at 3 months in terms of baseline characteristics. Finally, to provide data on an intention-to-treat basis, we conducted a sensitivity analysis using two scenarios (worst and best-case imputation), which were highly unrealistic.

Another possible limitation is that 2 procedures were used to record data at follow-up: visits to GPs and phone interviews. Phone interviews were used to decrease the number of patients lost to follow-up and were conducted by a trained research assistant. Outcome measures were easy to record by phone and the proportion of patients for whom data were collected by phone did not differ between the control and intervention groups. The difference in proportion of patients with persistent pain between phone and face-to-face interviews is probably a result of patient improvement. The patients without persistent pain were less likely to visit their doctors at 3 months than patients with persistent pain.

Only patients reporting persistent pain were reassessed at 3-month follow-up, which limits the ability of this study to detect effects of the intervention on outcome measures that may influence back-pain recurrence such as disability and fear-avoidance beliefs.

Finally, the sample size calculation did not take into account the cluster effect, which resulted in a lower power of our study, but which has no impact since the study results were positive.

### Overall evidence

Low-tech and easy-to-disseminate interventions have enormous promise in LBP interventions. The level of improvement described in this study is modest, but the cost and complexity of the intervention is minimal. Therefore, the implications and generalizability of this intervention are substantial.

## Supporting Information

Checklist S1 CONSORT Checklist
(0.04 MB DOC)

Protocol S1 Trial protocol
(2.15 MB PDF)

Alternate Language Abstract S1 Translation of the abstract into French by Serge Poiraudeau
(0.02 MB DOC)
