# Supplementary material for: Effect of a Simple Information Booklet on Pain Persistence after an Acute Episode of Low Back Pain: A Non-Randomized Trial in a Primary Care Setting
Source: PLoS One. 2007 Aug 8;2(8):e706. doi: 10.1371/journal.pone.0000706 (PMC1939729; doi:10.1371/journal.pone.0000706)
Supplement: Protocol S1 — Trial protocol (2.15 MB PDF) [file pone.0000706.s002.pdf]

Laboratoire Aventis

PROTOCOLE D'ETUDE OBSERVATIONNELLE AVEC RECUEIL DE DONNES DIRECTEMENT OU INDIRECTEMENT NOMINATIVES

D I S C O

EVALUATION DE L'IMPACT D'UN PROGRAMME EDUCATIF "LE GUIDE DU DOS" SUR L'EVOLUTION DES LOMBALGIES COMMUNES EN MEDECINE GENERALE

IXP 0001A / 9001

Expert scientifique:

Professeur Serge POIRAUDEAU
Hôpital Cochin
Service de Rééducation et Réadaptation
27, rue du Faubourg Saint-Jacques
75679 PARIS Cedex 14
Tel: 01.58.41.25.49
e-mail: serge.poiraudeau@cch.ap-hop-paris.fr

Promoteur de l'étude :

Laboratoire Aventis
46, quai de la Rapée
75601 Paris Cedex 12

Signataires du protocole:

- **Chef de Projet**

Fernand CORIAT
Laboratoire Aventis
Direction Médicale
Unité Thérapeutique Antalgie
46, quai de la Rapée
75601 PARIS Cedex 12
Tel: 01.55.71.52.91
e-mail: fernand.coriat@aventis.com

- **Statisticien de l'étude**

Sylvie BRIN
Laboratoire Aventis
Direction Médicale
46, quai de la Rapée
75601 PARIS Cedex 12

Coordonnateur de l'étude:

Christine JUQUELIER
Laboratoire Aventis
Direction des Opérations Cliniques
46, quai de la Rapée
75601 PARIS Cedex 12
Tel: 01.55.71.53.31
e-mail: christine.juquelier@aventis.com

Prestataire de service:

MediSCAN – I.B.S
66, rue Sébastien MERCIER
75015 PARIS

Dates du protocole final et des versions ultérieures : **28 mars 2003**

Date : 28 mars 2003 **Document n°3**

Confidentiel - Ce document est la propriété du Laboratoire Aventis.
Toute partie de ce document ne peut être reproduit, utilisé ou modifié
sans l'accord préalable du Laboratoire Aventis

SOMMAIRE

1. INTRODUCTION ET JUSTIFICATION DE L'ETUDE 10

2. OBJECTIFS DE L'ETUDE 12
2.1 Objectif principal 12
2.2 Objectif secondaire 12

3. METHODOLOGIE 12
3.1 Plan expérimental 12
3.2 Durée de l'étude et dates 13
3.3 Comités impliqués dans la conduite de l'étude 13

4. SELECTION DES CENTRES ET DES SUJETS 13
4.1 Sélection des centres 13
4.1.1 Méthode de sélection des centres 13
4.1.2 Nombre de centres 14
4.2 Sélection des sujets 14
4.2.1 Méthode de sélection des sujets 14
4.2.2 Nombre de sujets 14
4.2.3 Critères d'inclusion 14
4.2.4 Critères de non-inclusion 14

5. DEROULEMENT PRATIQUE DE L'ETUDE 15
5.1 Liste des données recueillies 15
5.1.1 Données du Médecin Observateur 15
5.1.2 Données du sujet 15
5.2 Déroulement de l'étude 16
5.3 Mode de recueil des données 17
5.4 Gestion des observations incomplètes 17

6. NOTIFICATION DES EFFETS INDESIRABLES 17

7. STATISTIQUE 17
7.1 Variables d'analyse 17
7.2 Populations étudiées 18
7.3 Méthodes d'analyse statistique 18
7.4 Analyse intermédiaire 19
7.5 Justification du nombre de sujets / de centres 19
7.6 Limites de l'étude 20
7.7 Généralisation des conclusions 20

8. CONSIDERATIONS ETHIQUES ET LEGALES 20
8.1 Cadre réglementaire de l'étude 20
8.2 Délégation des tâches par le Médecin Observateur 20
8.3 Information des sujets 20
8.4 Protection des données personnelles 21
8.4.1 Concernant le sujet 21
8.4.2 Concernant le Médecin Observateur 21

8.5 Soumission du protocole, de ses éventuels amendements et du contrat d'étude 21
8.6 Amendement(s) au protocole et approbation de(des) l'amendement(s) 21
8.7 Arrêt de l'étude 21
8.8 Contrôle qualité des données 22
8.9 Archivage 22
8.10 Responsabilité et assurance du promoteur 22

9. DOCUMENTATION ET UTILISATION DES RESULTATS DE L'ETUDE 22
9.1 Documentation de l'étude 22
9.2 Utilisation des résultats de l'étude 23

10. ENGAGEMENTS DU PROMOTEUR ET DU MEDECIN OBSERVATEUR 24
10.1 Engagement du promoteur 24
10.2 Engagement du Médecin Observateur 25

11. REFERENCES 26

ANNEXES 28

COMITE SCIENTIFIQUE

Professeur Serge POIRAUDEAU
Hôpital Cochin
Service de Rééducation et Réadaptation
27, rue du Faubourg Saint-Jacques
75679 PARIS Cedex 14
Tel: 01.58.41.25.49
e-mail: serge.poiraudeau@cch.ap-hop-paris.fr

Docteur Emmanuel COUDEYRE
Hôpital Lapeyronie
Service Central de Rééducation Fonctionnelle
371, avenue du Doyen Giraud
34295 MONTPELLIER Cedex 5
Tel: 04.67.33.87.11
e-mail: e-coudeyre@chu-montpellier.fr

COMITE DE PILOTAGE

EXPERTS SCIENTIFIQUES

Professeur Serge POIRAUDEAU
Hôpital Cochin
Service de Rééducation et Réadaptation
27, rue du Faubourg Saint-Jacques
75679 PARIS Cedex 14
Tel: 01.58.41.25.49
e-mail: serge.poiraudeau@cch.ap-hop-paris.fr

Docteur Emmanuel COUDEYRE
Hôpital Lapeyronie
Service Central de Rééducation Fonctionnelle
371, avenue du Doyen Giraud
34295 MONTPELLIER Cedex 5
Tel: 04.67.33.87.11
e-mail: e-coudeyre@chu-montpellier.fr

CHEF DE PROJET

Fernand CORIAT
Laboratoire Aventis
Unité Thérapeutique Antalgie
Direction Médicale
46, quai de la Rapée
75601 PARIS Cedex 12
Tel: 01.55.71.52.91
e-mail: fernand.coriat@aventis.com

STATISTICIEN

Sylvie BRIN
Laboratoire Aventis
Direction Médicale
46, quai de la Rapée
75601 PARIS Cedex 12
Tel: 01.55.71.03.37
e-mail: sylvie.brin@aventis.com

RESUME DU PROTOCOLE

Code de l'étude
IXP 0001A / 9001

Titre
Evaluation de l'impact d'un programme éducatif "Le Guide du Dos" sur l'évolution des Lombalgies communes en Médecine Générale.

Objectifs

- **Objectif principal:**

Evaluer l'impact d'un programme éducatif par l'information écrite "Le Guide du Dos" sur le passage à la chronicité de la lombalgie commune, en plus de l'information orale habituelle délivrée à l'ensemble des sujets lombalgiques.

- **Objectif secondaire:**

Evaluer l'impact de l'information orale et/ou écrite "Le Guide du dos" sur:
1. Les croyances, l''appréhension et les conduites d'évitement liées à l'activité physique.
2. L'incapacité fonctionnelle.
3. L'intensité de la douleur.
4. La satisfaction en rapport avec l'information reçue.
5. L'activité (arrêt de travail et reprise d'activité).
6. La prise en charge thérapeutique (prescriptions de traitements, visites spécialisées, examens complémentaires).

Méthodologie

- **Plan expérimental**

Etude observationnelle nationale, multicentrique de type "ici-ailleurs"; sur l'évaluation d'un programme éducatif. Cette étude permettra de comparer au même moment 2 communautés de "Sujets", distinctes géographiquement, dont une reçoit le "Guide du dos" et l'autre pas.

Les sujets inclus dans le groupe témoin "groupe ici" recevront une information orale non-standardisée de la part de leur Médecin Généraliste conformément à ses pratiques.

Les sujets inclus dans le "groupe ailleurs" recevront en plus de l'information orale non-standardisée de la part de leur Médecin Généraliste conformément à ses pratiques, un livret d'information "Le Guide du dos"* remis à l'issue de leur 1ère consultation après les avoir informés de son contenu, de son l'intérêt et de la manière de l'utiliser.

*Le "Guide du dos", est la version française du "Back Book". C'est un livret d'information et de conseils les plus judicieux et les plus récents destinés aux sujets lombalgiques pour gérer leur douleur dés qu'elle se manifeste, éviter d'être trop gêné et récupérer une mobilité plus rapidement. Ce guide est le seul livret en français dont le contenu soit validé et conformes aux données les plus récentes sur les recherches médicales.

- **Durée de l'étude et calendrier**

L'étude sera proposée aux Médecins observateurs en septembre 2003.

- Début des inclusions:	septembre 2003
- Fin des inclusions:	octobre 2003
- Fin du suivi:	février 2004
- Durée de l'étude:	6 mois

- **Recrutement des centres**

Multicentrique et national.
1800 Médecins Généralistes sélectionnés ayant accepté de participer à l'étude. Chaque Médecin Observateur devant inclure 4 sujets.

Le recrutement national des Médecins Généralistes sera effectué par tirage au sort à partir du fichier LOGIMED des Médecins Généralistes français ayant un potentiel suffisant pour inclure en 1 mois 4 sujets présentant une lombalgie aiguë commune.

Ce recrutement sera réalisé par stratification géographique (30 régions) et la représentativité des médecins sera assurée par la sélection aléatoire.

La France sera divisée en 30 régions géographiques selon le découpage régional du Laboratoire Aventis. Ces régions seront divisées en 2 groupes, le groupe "Témoin" et le groupe "Guide du dos". Chaque groupe sera constitué de 15 régions et chacune des régions sera composée de 60 Médecins Généraliste tirés au sort et ayant acceptés de participer à l'étude.

➢ **Recrutement des sujets**

Inclure 7200 sujets pour obtenir 6120 sujets évaluables à la fin de la phase 3 de l'étude.

Critères d'inclusion

- Sujets âgés de plus de 18 ans.
- Sujets en activité professionnelle.
- Sujets présentant une lombalgie aiguë commune (définie comme une douleur d'intensité modérée de la région lombaire n'irradiant pas au-delà du pli fessier), évoluant depuis moins de 8 jours.
- L'intensité de la douleur mesurée sur une Echelle Numérique de 0 "pas de douleur" à 10 "douleur maximale imaginable" devra être comprise entre 3 et 10 bornes incluses.
- Sujets vus pour la première fois pour cet épisode de lombalgie aiguë commune.
- Sujets venant en consultation au cabinet ou vus à domicile.

Critères de non-inclusion

- Sujets ayant présenté un épisode de lombalgie aiguë commune dans l'année précédant l'épisode actuel ou ayant déjà consulté pour ce même épisode.
- Litige médico-légal en cours.
- Lombalgies potentiellement liées à une pathologie infectieuse, tumorale ou inflammatoire (SPA,...).
- Antécédents d'intervention chirurgicale sur le rachis lombaire.
- Lombalgie aiguë commune avec irradiation au-delà du pli fessier.
- Grossesse connue du Médecin.
- Sujets ne pouvant pas être contactés ou vus après le 3^{éme} mois suivant son inclusion.
- Sujets dans l'incapacité mentale de comprendre et/ou de se soumettre à l'étude.
- Sujets dans l'incapacité de lire et comprendre un document écrit en français.
- Sujets sans activité professionnelle.

Déroulement de l'étude

L'étude sera réalisée en 3 phases:

- **Phase 1: Lors de la mise en place de l'étude**

  Une fiche de recueil d'information médecin sera remplie par le Médecin Observateur sélectionné.

- **Phase 2: Inclusion des sujets lors d'une consultation ou d'une visite (V.1)**

  Une fiche de recueil d'information sujet sera remplie par le Médecin Observateur.
  Une information orale et/ou écrite " Le Guide du Dos" sur la prise en charge de leur mal de dos sera délivrée aux sujets par les Médecins Observateurs selon leur groupe d'appartenance groupe "Témoin" ou groupe "Guide du dos".

- **Phase 3: Suivi des sujets lors d'une consultation ou d'une visite ou d'un appel téléphonique (au-delà du 3ème mois – V2)**

  Une fiche de recueil d'information sujet sera remplie par le Médecin Observateur à partir du troisième mois suivant l'inclusion.

Données recueillies

Les principales données seront recueillies à l'aide de questionnaires indirectement nominatif.

- **Phase 1:** Phase transversale
  - **Pour les Médecins Observateurs:**
    - ✓ Les caractéristiques démographiques.
    - ✓ Les données sur le mode d'exercice.
    - ✓ Les antécédents personnels en rapport avec la lombalgie.
    - ✓ La place de l'information au sein de la consultation.
    - ✓ Les données sur les habitudes de prescription, de mise au repos, d'activités physique et professionnelle tant dans la prise en charge de la lombalgie commune que de la lombalgie chronique.
    - ✓ Leurs opinions concernant les relations entre activité physique et lombalgie.
- **Phase 2**: Phase transversale
  - **Pour les sujets (V.1):**
    - ✓ Les caractéristiques démographiques.
    - ✓ Les données en rapport avec la lombalgie et en particulier l'avis du sujet sur la relation entre l'activité professionnelle et physique sur la lombalgie commune.
- **Phase 3**: Phase longitudinale
  - **Pour les sujets (au-delà du 3ème mois – V2) :**

Tous les sujets ayant participé à la phase 2 de l'étude pourront participer à la phase 3.

- ✓ Seront renseignés, en plus des données en rapport avec la lombalgie, les éventuels arrêts et durée des arrêts de travail, la reprise du travail pendant cette période et la prise en charge thérapeutique.
- ✓ La satisfaction en rapport avec l'information reçue.

Méthodes statistiques

L'évaluation de l'impact du programme éducatif par l'information orale et/ou écrite "Le Guide du Dos" sera mesurée par le pourcentage de sujets passant à la chronicité.

Le passage ou non à la chronicité sera défini de la façon suivante (à partir des données recueillies lors de l'évaluation V2 au-delà du 3ème mois).

- **Passage à la chronicité de la lombalgie commune :**
  - Réponse **« oui »** à la question : « votre douleur du dos persiste t-elle depuis la première consultation de manière quasi quotidienne ».
  - **Et** réponse **« non »** à la question « la douleur irradie t-elle au delà du genou ».
- **Non-passage à la chronicité de la lombalgie commune :**
  - Réponse **« non »** à la question : « votre douleur du dos persiste t-elle depuis la première consultation de manière quasi quotidienne ».

    Ou
  - Réponse **« oui »** à la question : « votre douleur du dos persiste t-elle depuis la première consultation de manière quasi quotidienne ».
  - **Et** réponse **«oui »** à la question « votre douleur irradie t-elle au-delà du genou ».

Analyse du critère principal

La mesure de l'impact du programme éducatif par l'information orale et/ou écrite "Le guide du Dos" délivré par le Médecin Observateur sur le passage à la chronicité de la lombalgie commune, sera effectuée à partir de la comparaison du pourcentage de sujets passés à la chronicité entre le groupe "Témoin" et le groupe "Guide du dos". Un test du Chi2 sera effectué au seuil α= 5%.

Les sujets recrutés dans le groupe "Témoin" et le groupe "Guide du dos" pouvant ne pas être comparables, des ajustements sur des facteurs de confusion, tels que le sexe, l'âge et le niveau d'étude, seront alors envisagés. En effet, le choix des régions "Témoin" et "Guide du dos" ayant été effectué de façon arbitraire (région avec numéro impair : région "Témoin » et région avec numéro pair : région "Guide du dos".

ABREVIATIONS ET DEFINITIONS

- **ANAES** **Agence Nationale d'Accréditation et d'Evaluation en Santé.**
- **CNOM** : Conseil National de l'Ordre des Médecins.
- **CNIL** : Commission Nationale de l'Informatique et des Libertés.
- **FABQ** : Fear Avoidance Belief Questionnaire.
  Echelle explorant les facteurs psycho-sociaux prédictifs du passage à la chronicité de la lombalgie aiguë en 11 questions.
- **QUEBEC :** Echelle explorant le handicap lié à la lombalgie commune en 20 questions.
- **EN** Echelle Numérique de 0 à 10.
- **EVS** Echelle Verbale en 4 points.
- **V1** Visite d'inclusion du sujet.
- **V2** Visite de suivi du sujet au-delà du 3éme mois.

1. INTRODUCTION ET JUSTIFICATION DE L'ETUDE

La lombalgie commune définie comme une douleur de la région lombaire n'irradiant pas au-delà du pli fessier[1] est une pathologie fréquente et constitue un véritable problème de santé publique dans les pays développés.

Une étude scandinave[2] a permis d'évaluer la prévalence de la lombalgie au cours de la vie dans les pays nordiques à 60-65 %. Cet ordre de grandeur est comparable pour le Royaume Uni[3].
Pour la France, un rapport de 1994 du Haut Comité de la santé publique[4], a estimé que 52 actes sur 1 000 de médecins généralistes concernaient la pathologie rachidienne. Si dans la grande majorité des cas la lombalgie commune évolue de façon favorable, c'est la minorité de patients lombalgiques (6 à 8%) évoluant vers la chronicité[5] qui est responsable de la majeure partie des coûts de santé liées à cette pathologie[6]. Ce stade est défini par une douleur habituelle de la région lombaire évoluant depuis plus de 3 mois. Cette douleur peut s'accompagner d'une irradiation à la fesse, à la crête iliaque, voire à la cuisse et ne dépasse qu'exceptionnellement le genou (accord professionnel)[7], ceci avec souvent des périodes sans douleur.

Les facteurs responsables du passage à la chronicité de la pathologie lombaire commune sont pour une grande part des facteurs sociaux, culturels et environnementaux[8,9]. Parmi ces facteurs les comportements d'appréhension conduisant à des conduites d'évitement vis à vis de l'activité physique sont majeurs. Plusieurs échelles d'évaluation de ces peurs et croyances ont été développées parmi lesquelles, seul le Fear Avoidance Belief Questionnaire (FABQ)[10] est validé en français.

Améliorer l'information initiale des patients lombalgiques peut permettre de modifier leurs connaissances et secondairement leur attitude face aux douleurs lombaires. Cette amélioration peut passer par une meilleure communication orale mais elle peut également être favorisée par l'utilisation de documents écrits standardisés conformément aux recommandations de l'ANAES[11].

Différents travaux ont étudié l'impact d'une information ciblée sur l'évolution de la lombalgie. L'étude de Burton[12] a souligné l'intérêt d'un simple livret, celle de Buchbinder[13] a mis en évidence l'efficacité d'une campagne médiatique de grande envergure. Ces deux études ont évalué l'impact de l'information sur des patients lombalgiques suivis en ville et ont montré l'intérêt d'une information ciblée sur le passage à la chronicité et sur la consommation médicale.

Aucune étude de ce type n'a été menée en France à ce jour. Par conséquent la qualité du message délivré par les médecins généralistes lors de la prise en charge d'une lombalgie aiguë sur le territoire français n'est pas connue de façon précise. Il est pertinent d'évaluer la qualité de cette information quelle soit orale et/ou écrite ainsi que son impact.

L'objectif de cette étude observationnelle est d'évaluer l'impact de l'information orale et/ou écrite reçue par les patients souffrant de lombalgie aiguë lors de leur prise en charge initiale en Médecine Générale. La méthodologie utilisée sera de type "ici-ailleurs", elle consiste à comparer, au même moment, des communautés distinctes géographiquement dont l'une reçoit l'intervention et l'autre pas, conformément aux travaux menés dans l'évaluation des actions de Santé Publique[14].

L'intervention consistera pour la moitié des Médecins Généralistes sélectionnés à délivrer une information orale non-standardisée conformément à leur pratique habituelle, pour l'autre moitié des praticiens en plus de l'information orale ils remettront un livret d'information le "Guide du dos" à leurs patients. Le "Guide du dos"[15], version française du "Back Book" est le seul livret d'information en français destinés aux sujets lombalgiques dont le contenu soit validé et conformes aux données les plus récentes de la littérature.

Ce "Guide du dos" est un livret d'information basé sur des conseils les plus judicieux et les plus récentes découvertes médicales sur la prise en charge du mal de dos. Conçu à l'intention des sujets lombalgiques, il a été rédigé afin de répondre à une forte demande d'information pour gérer la douleur dés qu'elle se manifeste, éviter d'être trop gêné et récupérer une mobilité plus rapidement.

Mille huit cent Médecins Généralistes seront sélectionnés par tirage au sort aléatoire sur la France découpée en 30 régions géographiques[Annexes1;2l] afin d'inclure 7200 sujets présentant une lombalgie aiguë commune définie comme une douleur d'intensité modérée de la région lombaire n'irradiant pas au-delà du pli fessier, évoluant depuis moins de 8 jours. L'intensité de la douleur sera mesurée sur une Echelle Numérique (EN) de 0 "pas de douleur" à 10 "douleur maximale imaginable" et devra être comprise entre 3 et 10 bornes incluses[16,17].

L'étude se déroulera sur six mois pour permettre à chaque Médecin Observateur d'inclure quatre patients en 1 mois est d'être à même de les suivre pendant au moins trois mois (cf passage à la chronicité).

Le critère principal étudié est le passage à la chronicité c'est à dire la persistance d'une douleur trois mois après le début de l'épisode ayant conduit à consulter un Médecin Généraliste. Les croyances, l'appréhension et les conduites d'évitement liées à l'activité seront également évaluées ainsi que l'incapacité fonctionnelle.

Il s'agit d'une étude observationnelle ne modifiant pas la prise en charge médicale habituelle des personnes entrant dans l'étude, ne portant pas atteinte à l'intégrité physique ou psychique et ne nécessitant pas de visite particulière de suivi pour les personnes entrant dans l'étude. Aucune procédure supplémentaire de diagnostic ou de surveillance ne sera appliquée aux sujets.

Des données indirectement nominatives (numéro du sujet) au sens de la loi Informatique et Libertés et concernant les sujets seront recueillies par le Médecin Observateur. Le recueil de ce type de données se justifie par le niveau qualitatif attendu pour l'étude.

2. OBJECTIFS DE L'ETUDE

2.1 Objectif principal

L'objectif principal de cette étude est d'évaluer l'impact d'un programme éducatif par l'information écrite "Le Guide du Dos" sur le passage à la chronicité de la lombalgie commune, en plus de l'information orale habituelle délivrée à l'ensemble des sujets lombalgiques

2.2 Objectif secondaire

L'objectif secondaire de cette étude est d'évaluer l'impact de l'information orale et/ou écrite"Le guide du dos" sur:

1. Les croyances, l'appréhension et les conduites d'évitement liées à l'activité physique.
2. L'incapacité fonctionnelle.
3. L'intensité de la douleur.
4. La satisfaction en rapport avec l'information reçue.
5. L'activité (arrêt de travail et reprise d'activité).
6. La prise en charge thérapeutique (prescriptions de traitements, visites spécialisées, examens complémentaires).

3. METHODOLOGIE

3.1 Plan expérimental

Cette étude observationnelle nationale, multicentrique de type "ici-ailleurs"[(14)] sur l'évaluation d'un programme éducatif permettra de comparer au même moment 2 communautés de "Sujets", distinctes géographiquement, dont une reçoit le "Guide du dos" et l'autre pas.

Les sujets inclus dans le groupe témoin "groupe ici" recevront une information orale non-standardisée de la part de leur Médecin Généraliste conformément à ses pratiques.

Les sujets inclus dans le "groupe ailleurs" recevront en plus de l'information orale non-standardisée de la part de leur Médecin Généraliste conformément à ses pratiques un livret d'information "Le Guide du dos" remis à l'issue de leur 1ère consultation après les avoir informés de son contenu, de son l'intérêt et de la manière de l'utiliser.

La France sera divisée en 30 régions géographiques selon le découpage régional du Laboratoire Aventis[(Annexe 1)]. Ces régions seront divisées en 2 groupes, pour des raisons de compréhension le "groupe ici" constitué des sujets qui recevront une information orale non-standardisée de la part de leur Médecin Généraliste conformément à ses pratiques s'appellera le groupe "Témoin" et le"groupe ailleurs" constitué des sujets qui recevront en plus de l'information orale non-standardisée de la part de leur Médecin Généraliste conformément à ses pratiques un livret d'information "Le Guide du dos*" remis à l'issue de leur consultation s'appellera groupe "Guide du dos".

*Le Guide du dos[(15)] est la version française du "Back Book". C'est un livret d'information et de conseils les plus judicieux et les plus récents destinés aux sujets lombalgiques pour gérer leur douleur dés qu'elle se manifeste, éviter d'être trop gêné et récupérer une mobilité plus rapidement. Ce guide est le seul livret en français dont le contenu soit validé et conformes aux données les plus récentes sur les recherches médicales.

Chaque groupe sera constitué de 15 régions et chacune des régions sera composée de 60 Médecins Généraliste tirés au sort ayant acceptés de participer à l'étude.

La répartition selon les 2 groupes se fera de la façon suivante:

- Régions impaires[(Annexe 2)] composeront le groupe "Témoin"
- Régions paires[(Annexe 2)] composeront le groupe "Guide du dos"

Les Médecins Observateurs rempliront un formulaire de recueil des données les concernant ainsi que deux formulaires de recueil (J.1 et à partir de J.90) par sujet dans le cadre de l'étude.

Cette étude n'engendre aucune modification dans la prise en charge du sujet.

3.2 Durée de l'étude et dates

La durée prévue de l'étude est de 6 mois avec un recrutement des sujets débutant en septembre 2003 et finissant en octobre 2003 soit 1 mois. La durée totale de l'étude ou la période de recrutement des pourront varier.

- Début des inclusions: septembre 2003
- Fin des inclusions: octobre 2003
- Fin du suivi: février 2004
- Durée de l'étude: 6 mois

3.3 Comités impliqués dans la conduite de l'étude

3.3.1 Comité Scientifique

En amont de l'étude, un Comité Scientifique a été nommé spécifiquement afin de conseiller le Laboratoire Aventis et de valider l'ensemble du projet, à savoir :

- La pertinence scientifique de l'étude et ses objectifs.
- Les documents de recueil des données.
- Les procédures de contrôle de qualité des données en cours d'étude.

Le Comité Scientifique sera consulté par le Laboratoire Aventis pour toute question médicale ou scientifique survenant en cours d'étude.

Il conseillera le Laboratoire Aventis sur l'exploitation des données et la publication des résultats

3.3.2 Comité de Pilotage

Un Comité de Pilotage composé des membres du Comité Scientifique, du Chef de Projet et du Statisticien de l'étude gérera au quotidien toutes questions relatives à l'étude (scientifiques, logistiques etc..) sera le signataire de tous documents d'informations remis ou envoyés aux Médecins Observateurs et sera le garant de sa réalisation dans le cadre et en accord avec la réglementation française.

4. SELECTION DES CENTRES ET DES SUJETS

4.1 Sélection des centres

4.1.1 Méthode de sélection des centres

La représentativité de l'échantillon des centres sera assurée par un tirage au sort, avec stratification géographique, à probabilité égale et sans remise, réalisé à partir d'un fichier LOGIMED comportant l'ensemble des 22 000 Médecins Généralistes français ayant une activité libérale et un potentiel suffisant de sujets pour inclure en 1 mois 4 sujets présentant une lombalgie commune comme définie par ce protocole. *(voir paragraphe 4.2.3 critères d'inclusion et 4.2.4 critères de non-inclusion)*

La France sera divisée en 30 régions géographiques selon le découpage régional de la Visite Médicale *(voir chapitre 3.1 plan expérimental et annexe 1).* Ces régions seront divisées en 2 groupes, le groupe "Témoin" et le groupe "Guide du dos". Chaque groupe sera constitué de 15 régions et chacune des régions sera composée de 60 Médecins Observateurs.

Pour cela, après tirage au sort, une première liste de 60 Médecins Généralistes par région sera définie. Ces Médecins Généralistes seront contactés afin de leur proposer de participer à l'étude jusqu'à constitution d'une liste finale de 60 Médecins Observateurs.

Au regard des difficultés du recrutement des Médecins Observateurs nous constituerons une seconde liste de 60 autres Médecins Généralistes tirés au sort (à partir de la même source: Fichier LOGIMED) pour pallier les refus de participation des Médecins Généralistes de la première liste. Les éventuels refus seront renseignés.

4.1.2 Nombre de centres

Comme calculé au Chapitre 7.5 «Justification du nombre de sujets/de centres», 1800 Médecins généralistes seront sélectionnés et participeront à l'étude.
Chaque Médecin Observateur devant inclure au maximum 4 sujets.

4.2 Sélection des sujets

4.2.1 Méthode de sélection des sujets

Chaque Médecin Observateur devra inclure les 4 premiers sujets consécutifs, en activité professionnelle présentant les critères de sélection et venant en consultation au cabinet ou vus à domicile pour lombalgie aiguë commune.

4.2.2 Nombre de sujets

Comme calculé au Chapitre 7.5 «Justification du nombre de sujets/de centres », 7200 sujets seront inclus pour obtenir 6120 sujets évaluables à la fin de la phase 3 de l'étude.. Le nombre recommandé de sujets par centre est de 4.
Le recrutement des sujets pourra être interrompu dès que le nombre total de sujets nécessaires à l'étude sera atteint.

4.2.3 Critères d'inclusion

Les sujets remplissant tous les critères suivants seront envisagés pour l'inclusion dans l'étude :

- Sujets âgés de plus de 18 ans.
- Sujets en activité professionnelle.
- Sujets présentant une lombalgie aiguë commune (définie comme une douleur d'intensité modérée de la région lombaire n'irradiant pas au-delà du pli fessier), évoluant depuis moins de 8 jours.
- L'intensité de la douleur mesurée sur une Echelle Numérique de 0 "pas de douleur" à 10 "douleur maximale imaginable" devra être comprise entre 3 et 10 bornes incluses [16,17].
- Sujets vus pour la première fois pour cet épisode de lombalgie aiguë commune.
- Sujets venant en consultation au cabinet ou vu à domicile.

Une information détaillée sur le but de cette étude et ses modalités sera fournie au sujet avant le début de l'étude.

4.2.4 Critères de non-inclusion

Les sujets présentant un des critères suivants ne seront pas inclus dans l'étude :

- Sujets ayant présenté un épisode de lombalgie aiguë commune dans l'année précédant l'épisode actuel ou ayant déjà consulté pour ce même épisode.
- Litige médico-légal en cours.
- Lombalgies potentiellement liées à une pathologie infectieuse, tumorale ou inflammatoire (SPA,...).
- Antécédents d'intervention chirurgicale sur le rachis lombaire.
- Lombalgie aiguë commune avec irradiation au-delà du pli fessier.
- Grossesse connue du médecin.
- Sujets ne pouvant pas être contactés ou vus après le 3^{éme} mois suivant son inclusion.
- Sujets dans l'incapacité mentale de comprendre et/ou de se soumettre à l'étude.
- Sujets dans l'incapacité de lire et comprendre un document écrit en français.
- Sujets sans activité professionnelle.

Toute exception aux critères d'inclusion ou de non-inclusion doit être approuvée par le Comité de Pilotage, au cas par cas, avant l'inclusion dans l'étude. Ceci doit être documenté, par écrit, par le Responsable du Projet. Aucun sujet ne sera inclus dans l'étude plus d'une fois.

5. DEROULEMENT PRATIQUE DE L'ETUDE

5.1 Liste des données recueillies

5.1.1 Données du Médecin Observateur

Les médecins Généralistes devront être contactés dans l'ordre d'apparition du pré-échantillonnage par le personnel spécialement formé à cette étude observationnelle par le Laboratoire Aventis pour obtenir leur accord de participation et ceci, jusqu'à l'obtention dans chaque région géographique de 100% de l'effectif de médecins nécessaires

1800 Médecins Observateurs participeront à l'étude. Chaque Médecin Observateur inclura les 4 premiers sujets consécutifs consultant pour un épisode de lombalgie aiguë commune répondant aux critères d'inclusion et de non-inclusion.

Chaque Médecin Observateur remplira :

➢ Une fiche de recueil d'information Médecin comportant les données suivantes:

- Numéro de centre.
- Les données démographiques (âge, sexe), le nombre d'année d'exercice.
- Le secteur d'activité, la situation géographique.
- Antécédents personnels en rapport avec la lombalgie.
- Données sur la prise en charge des lombalgies.
- La place de l'information au sein de la consultation
- Un questionnaire concernant le lien entre les lombalgies et les activités physiques ou professionnelles.

Les Guides du dos seront fournis aux Médecins Observateurs appartenant aux régions paires groupe "Guide du dos"et ils seront remis systématiquement aux sujets inclus par ces Médecins Observateurs.

5.1.2 Données du sujet

Des données indirectement nominatives concernant les sujets seront recueillies dans le cadre de cette étude. Ces données seront les suivantes :

Chaque Médecin Observateur remplira :

1. **Une fiche de recueil d'information sujet à la Visite d'inclusion (V1),** comportant les données suivantes:
   - Date de la consultation/visite
   - Numéro du sujet.
   - Numéro de centre
   - Age, sexe.
   - Evaluation de l'intensité de la douleur (Echelle Numérique).
   - Evaluation du lien entre la douleur et l'activité professionnelle (Echelle Numérique).
   - Niveau d'étude.
   - Antécédents familiaux en rapport avec la lombalgie commune.
   - Données sur l'épisode actuel de lombalgie (date, traitements médicamenteux).
   - Un questionnaire explorant le handicap lié à la lombalgie commune (QUEBEC[(18)] version française)
   - Un questionnaire explorant les facteurs psycho-sociaux prédictifs du passage à la chronicité de la lombalgie commune. (FABQ[(10)] version française)
2. **Une fiche de recueil d'information sujet à la Visite de suivi V2 (au-delà du 3éme mois),** comportant les données suivantes:
   - Date de la consultation/visite/appel téléphonique
   - Numéro du sujet.

- Numéro de centre
- Moyen de recueil des données (consultation/visite/appel téléphonique)
- Données sur le suivi de l'épisode de lombalgie commune.(traitements médicamenteux, examens complémentaires, visites spécialisées).
- Evaluation de l'intensité de la douleur (Echelle Numérique).
- Activité Professionnelle ((Arrêt de travail, durée et reprise de travail)
- .Un questionnaire sur l'évaluation de l'information (orale et/ou écrite) reçue sur la prise en charge du mal de dos lors de l'inclusion.
- Evaluation de l'influence de l'information (orale et/ou écrite) sur l'évolution de l'épisode douloureux de la lombalgie commune (Echelle Verbale Simple).

Si la douleur est persistante, les questionnaires suivants seront à compléter:

- Un questionnaire explorant le handicap lié à la lombalgie commune (QUEBEC[(18)] version française)
- Un questionnaire explorant les facteurs psycho-sociaux prédictifs du passage à la chronicité de la lombalgie commune. (FABQ[(8)] version française)

5.2 Déroulement de l'étude

Le recrutement des Médecins Observateurs par le personnel spécialement formé à cette étude observationnelle par le Laboratoire Aventis, s'effectuera sur une période de 1 mois, celui de l'ensemble des sujets sur une période de 1 mois maximum, le suivi des sujets s'effectuera au 4^{éme} mois. La durée totale de cette étude observationnelle sera de 6 mois.

Le Médecin Généraliste sera considéré comme participant à cette étude observationnelle une fois le protocole et son contrat d'honoraires signés et reçus par à la société prestataire de services en charge de la logistique de l'étude.

5.2.1 Phase 1 et 2

Phases transversales de cette étude observationnelle en Médecine Générale. Elles viseront à décrire tant pour les Médecins Observateurs que pour les sujets les données démographiques, les caractéristiques actuelles de la lombalgie commune et les opinions concernant les relations entre activités physiques et professionnelles et la lombalgie commune.

5.2.1.1 Phase 1

Le Médecin Observateur devra:

- Compléter et signer les documents administratifs.
- Remplir la Fiche de recueil d'information Médecin.
- Adresser-le tout sous enveloppe T à la société prestataire de services en charge de la logistique de l'étude.

Pour le Médecin Observateur faisant parti du groupe "Guide du dos"

- Prendre connaissance du contenu du livret "Le Guide du dos".

5.2.1.2 Phase 2: Inclusion du sujet (V1)

Le Médecin Observateur devra:

- Vérifier les critères d'inclusion et de non-inclusion
- Informer le sujet de l'étude et lui remet la fiche d'Information Sujet.
- Compléter avec le sujet la Fiche de recueil d'information V1.
- Adresser l'exemplaire original de cette fiche sous enveloppe T à la société prestataire de services en charge de la logistique de l'étude.
- Dispenser une information orale non-standardisée conformément à sa pratique habituelle sur la prise en charge du mal du dos.

Pour le Médecin Observateur faisant parti du groupe "Guide du dos"

- Remettre aux sujets faisant parti du groupe "Guide du dos", le livret Le Guide du dos.
- Informer les sujets de son contenu, de son intérêt et de la manière de l'utiliser.

5.2.2 Phase 3

Au-delà du 3éme mois suivant les inclusions des sujets, le suivi sera réalisé soit lors d'une consultation ou d'une visite à domicile quelque qu'en soit le motif, soit en l'absence de celle-ci par un contact téléphonique au plus tard le 4ème mois (J.120).

Phase longitudinale de cette étude observationnelle. Elle visera à décrire chez les sujets l'évolution de leur lombalgie commune et leurs opinions concernant les relations entre activités physiques et professionnelles et la lombalgie commune ainsi que la satisfaction en rapport avec l'information orale et/ou écrite "Le Guide du dos" reçue dans le cadre de la prise en charge de leur mal de dos.

5.2.2.1 Phase3: Suivi du sujet V2 (au-delà du 3ème mois)

Le Médecin Observateur devra:

- Compléter avec le sujet la Fiche de recueil d'information sujet V2.
- Adresser l'exemplaire original de cette fiche sous enveloppe T à la société prestataire de services en charge de la logistique de l'étude.

5.3 Mode de recueil des données

Le recueil des données sur la Fiche d'information Médecin sera réalisée à l'aide d'un questionnaire spécifique. (cf paragraphe 5.1.1)

Le recueil des données sur les Fiches d'information Sujet à V1 et à V2 sera réalisé à l'aide de:

1. Questionnaires spécifiques (cf paragraphe 5.1.2)
2. Echelle Numérique de 0 à 10.
3. Echelle Verbale Simple en 4 points.
4. Questionnaire QUEBEC(18) en 20 questions.
5. Questionnaire FABQ(10) en 11 questions.

5.4 Gestion des observations incomplètes

Les observations incomplètes ne seront pas remplacées.

6. NOTIFICATION DES EFFETS INDESIRABLES

Le présent protocole n'a pas d'influence sur la prescription médicale. Cette étude ne dispense pas le Médecin Observateur de la déclaration au centre régional de pharmacovigilance dont il dépend (la liste complète de ces centres est disponible dans le dictionnaire Vidal) de tout effet indésirable grave ou inattendu potentiellement liés à des produits de santé.
Le Médecin Observateur pourra, s'il le souhaite, notifier au Laboratoire Aventis tout effet grave potentiellement lié à un produit commercialisé par le Laboratoire Aventis. Dans ce cas, le Médecin Observateur est invité à se mettre en contact avec le Responsable de l'étude au sein du Laboratoire Aventis qui lui précisera les modalités de notification de ces effets.

7. STATISTIQUE

7.1 Variables d'analyse

La variable d'analyse principale est évaluation de l'impact de l'information orale et /ou écrite "Le Guide du dos" délivrée par les Médecins Généralistes aux sujets lombalgiques, sur le passage à la chronicité de la lombalgie commune.

Cet impact sera mesuré par le pourcentage de patient passant à la chronicité.

Le passage ou non à la chronicité sera défini de la façon suivante (à partir des données recueillies lors de l'évaluation V2 au delà du 3ème mois):

➢ **Passage à la chronicité de la lombalgie commune:**

- Réponse **« oui »** à la question : « votre douleur du dos persiste t-elle depuis la première consultation de manière quasi quotidienne ».
- **Et** réponse **« non »** à la question « la douleur irradie t-elle au delà du genou ».

➢ **Non-passage à la chronicité de la lombalgie commune:**

- Réponse **« non »** à la question : « votre douleur du dos persiste t-elle depuis la première consultation de manière quasi quotidienne ».

Ou

- Réponse **« oui »** à la question : « votre douleur du dos persiste t-elle depuis la première consultation de manière quasi quotidienne ».
- **Et** réponse **«oui »** à la question « votre douleur irradie t-elle au-delà du genou ».

7.2 Populations étudiées

L'analyse du critère de jugement principal et l'analyse des critères de jugement secondaires seront effectuées

- Sur la population de sujets pour lesquels une évaluation au delà du 3ème mois a été possible (variables définissant le passage à la chronicité renseignées).
- Sur l'ensemble des sujets inclus. Il sera fait l'hypothèse suivante pour les sujets pour lesquels nous n'auront pu avoir les variables principales renseignées : ne pas revenir voir son Médecin Observateur impliquerait un non-passage à la chronicité.

Seule la population de Médecins Observateurs ayant inclus un sujet évaluable sera décrite.

Le nombre de Médecins Observateurs participant à l'étude sera présenté au global.

7.3 Méthodes d'analyse statistique

Les données quantitatives seront décrites par leur effectif, leur moyenne, leur écart type, leur médiane et leurs valeurs extrêmes. Les données qualitatives seront décrites par leur effectif et leur pourcentage. Les intervalles de confiance bilatéraux à 95% seront fournis lorsque cela sera pertinent.

7.3.1 Description de la population des Médecins Observateurs

Les informations décrites porteront sur :

- Les données démographiques (âge, sexe), nombre d'années d'exercice, secteur d'activité, situation géographique (rural, semi-rural, urbain). Ces données seront comparées à titre descriptif à celles de la population des Médecins Généralistes français en activité. Le nombre de médecins ayant refusé de participer à l'étude sera précisé.
- Les éventuels antécédents personnels de lombalgie concernant le Médecin Observateur et son vécu.
- La place de l'information au sein de la consultation.
- Les données sur le mode d'exercice, sur les habitudes de prescription, de mise au repos, d'activité physique et professionnelle tant dans la prise en charge de la lombalgie commune que de la lombalgie chronique, leurs opinions concernant les relations entre activités physiques et lombalgies.

7.3.2 Description de la population des sujets

Seront décrites pour chacun des deux groupes groupe "Témoin" et groupe "Guide du dos" et globalement:

- Les données démographiques.

- Les données en rapport avec la lombalgie et en particulier, l'avis du sujet sur la relation entre activité professionnelle et physique sur la lombalgie commune.
- Des données supplémentaires seront également recueillies lors de l'évaluation du sujet réalisée au-delà du 3ème mois et décrites selon les deux groupes et globalement :
  - ✓ Les éventuels arrêts et durée des arrêts de travail, la reprise du travail pendant cette période et la prise en charge thérapeutique.
  - ✓ La satisfaction en rapport avec l'information reçue.

7.3.3 Analyse du critère principal

La mesure de l'impact du programme éducatif par l'information orale et/ou écrite "Le Guide du dos" délivrée par le Médecin Observateur sur le passage à la chronicité de la lombalgie commune sera effectuée à partir de la comparaison du pourcentage de sujets passés à la chronicité entre le groupe "Témoin" et le groupe "Guide du dos". Un test du Chi2 sera effectué au seuil α= 5%.

Les sujets recrutés dans le groupe "Témoin" et le groupe "Guide du dos" pouvant ne pas être comparables, des ajustements sur des facteurs de confusion, tels que le sexe, l'âge et le niveau d'étude, seront alors envisagés. En effet, le choix des régions "Témoin" et "Guide du dos" ayant été effectué de façon arbitraire (région avec numéro impair : région "Témoin » et région avec numéro pair : région "Guide du dos".

7.3.4 Analyse du critère secondaire

La mesure de l'impact du programme éducatif par l'information orale et/ou écrite "Le Guide du dos" délivrée par le Médecin Observateur sur le passage à la chronicité de la lombalgie commune, sera effectuée à partir des comparaisons descriptives et en pourcentage entre le groupe "Témoin" et le groupe "Guide du dos".sur:

- L'évolution entre V1 et V2 du score sur l'incapacité fonctionnelle (QUEBEC[(18)])
- L'évolution entre V1 et V2 du score portant sur les peurs et croyances concernant le rachis lombaire (FABQ[(10)]).
- L'évolution entre V1 et V2 de l'intensité de la douleur (E.N)
- En pourcentage à V2 de la satisfaction des sujets en rapport avec l'information reçue (EVS)
- En pourcentage à V2 sur l'activité professionnelle (arrêts de travail et reprise d'activité).
- L'évolution entre V1 et V2 de la prise en charge thérapeutique (prescriptions médicamenteuses).
- Le pourcentage à V2 de visites spécialisées.
- Le pourcentage à V2 d'examens complémentaires.

7.4 Analyse intermédiaire

Aucune analyse intermédiaire n'est prévue pour cette étude.

7.5 Justification du nombre de sujets / de centres

7.5.1 Nombre de sujets

La variable d'analyse principale est le pourcentage, évalué à la visite V2 de sujets passant d'une lombalgie commune à une lombalgie chronique. Selon les études[(5)], ce pourcentage varie de 6 à 8%.
En considérant que ce pourcentage pourrait être de 7% dans le groupe "Témoins" et de 5% dans le groupe "Guide du dos", 6120 sujets (3060 sujets par groupe) sont nécessaires pour mettre en évidence une telle différence avec un seuil de signification de 5% (test bilatéral) et une puissance de 90%. Afin de tenir compte de sujets non-évaluables (15%), 7200 sujets au total devront être inclus.

7.5.2 Nombre de centres

Compte tenu des contraintes de l'étude, des capacités de recrutement par centre observateur sur la période retenue par le protocole de l'étude, il a été décidé de demander à chaque Médecin Observateur d'inclure 4 sujets.
Pour inclure ces 7200 patients, il est estimé que 1800 Médecins Observateurs seront nécessaires dans cette étude.

7.6 Limites de l'étude

Le tirage au sort devrait assurer la représentativité de l'échantillon, mais le médecin pouvant refuser de participer à l'étude ou le médecin ne pouvant inclure les sujets demandés, l'extrapolation des résultats devra être prudente.

7.7 Généralisation des conclusions

Le tirage au sort doit assurer la représentativité de l'échantillon. Les conclusions de cette étude pourront être extrapolées à la prise en charge des patients lombalgiques sur le territoire français

8. CONSIDERATIONS ETHIQUES ET LEGALES

8.1 Cadre réglementaire de l'étude

Il s'agit d'une étude observationnelle ne modifiant pas la prise en charge médicale habituelle des sujets entrant dans l'étude, ne portant pas atteinte à leur intégrité physique ou psychique et ne nécessitant pas de visite particulière de suivi pour ces sujets. Dans ces conditions cette étude ne s'inscrit pas dans le champ d'application des articles L.1121-1 et suivants du Code de la Santé Publique.

En outre, le protocole de l'étude prévoit le recueil de données directement ou indirectement nominatives concernant les sujets ainsi que les Médecins Observateurs

Le recours à des données nominatives se justifie par le niveau qualitatif attendu pour l'étude. En effet le caractère nominatif des données enregistrées est nécessaire pour :

- Assurer un meilleur suivi des sujets par chaque Médecin Observateur,
- Assurer une meilleur traçabilité des données des sujets par le Laboratoire Aventis
- Permettre d'éventuels audits des centres des Médecins Observateurs.

De ce fait, l'étude sera conduite conformément à la loi "Informatique et Libertés" relative au traitement de données nominatives dans le domaine de la santé (loi n°94548 du 1er juillet 1994 modifiant la loi du 06 janvier 1978 et son décret d'application du 09 mai 1995).

8.2 Délégation des tâches par le Médecin Observateur

Aucune délégation de tâches ne sera autorisée pour cette étude.

8.3 Information des sujets

Cette étude prévoit le recueil de données nominatives (directement ou indirectement) au sens de la loi "Informatique et Libertés". De ce fait, et conformément à cette loi, le sujet devra avoir été informé par le Médecin Observateur et avant d'entrer dans l'étude, sous une forme compréhensible par lui, de la nature, de l'objectif et des conséquences possibles de l'étude, du recueil de données nominatives (directement ou indirectement) ainsi que de l'existence d'un traitement automatisé de ces données, des finalités de ce recueil et de son droit d'accès et de rectifications. Un formulaire d'information (annexe 3) sera remis à chaque sujet participant par le Médecin Observateur. Ce formulaire contient tous les éléments requis par la loi française et les directives du Laboratoire Aventis.

8.4 Protection des données personnelles

8.4.1 Concernant le sujet

Le nom, les initiales des sujets ne seront pas révélés au promoteur ou à son représentant, seul le Médecin Observateur possédera une liste de correspondance initiales du sujet et numéro d'observation. Seuls le numéro et les données démographiques du sujet apparaîtront dans les fiches de recueil des données. Ces données seront maintenues par le Laboratoire Aventis ou son représentant dans une base de données informatique conformément aux obligations définies par la loi "Informatique et Libertés". Tout document, quelque qu'il soit, transmis au promoteur ou à son représentant ne comportera qu'un numéro d'observation. Seules des données anonymes et résumées seront communiquées dans le cadre de l'analyse statistique.

8.4.2 Concernant le Médecin Observateur

Les données nominatives des Médecins Observateurs seront recueillies et maintenues par le Laboratoire Aventis ou son représentant dans une base de données conformément à la loi "Informatique et Libertés".

Dans le cadre de cette étude, des données concernant le Médecin Observateurs, autres que des données administratives, seront recueillies par le Laboratoire Aventis ou son représentant. Le Médecin Observateur est informé par le présent protocole (chapitre 5.1.1) de la nature des données recueillies le concernant. Pour ces données, le Médecin Observateur pourra exercer à tout moment son droit d'accès et de rectification.

8.5 Soumission du protocole, de ses éventuels amendements et du contrat d'étude

Avant le début de l'étude, le protocole d'étude, le formulaire d'information, et tous les autres documents utiles seront soumis aux autorités compétentes, conformément à la loi française, et notamment, l'avis du Comité Consultatif sur le Traitement de l'Information en Matière de Recherche dans le Domaine de la Santé, ainsi que l'autorisation de la Commission Nationale de l'Informatique et des Libertés devront avoir été obtenus avant l'initiation de l'étude.
Avant l'inclusion du premier sujet dans l'étude, toutes les obligations éthiques et réglementaires doivent être respectées.
Le médecin observateur doit conserver une trace de toutes les communications avec les autorités.

Conformément à l'article L.4113.6 du Code de la Santé Publique, les médecins étant rémunérés ou recevant des avantages en nature ou en espèces, le protocole et le contrat d'étude devront avoir été soumis au Conseil de l'Ordre National des Médecins, et avoir reçu un avis favorable avant la mise en place de l'étude.

8.6 Amendement(s) au protocole et approbation de(des) l'amendement(s)

Ni le Médecin Observateur ni le promoteur ne modifieront ce protocole d'étude sans obtenir l'accord écrit de l'autre partie. Après le début de l'étude, les amendements ne doivent être faits que dans des cas exceptionnels. Les modifications deviennent alors partie intégrante du protocole de l'étude.
Tout amendement au protocole ultérieur aux dépôts aux autorités compétentes sera soumis à ces même autorités, conformément à la loi française.

8.7 Arrêt de l'étude

Le promoteur ou le Médecin Observateur pourra interrompre l'étude à tout moment. Cette interruption devra avoir lieu si possible après concertation mutuelle.
Tout le matériel ayant trait à l'étude devra être retourné, détruit ou conservé, selon les directives du promoteur.

8.8 Contrôle qualité des données

Pour cette étude, aucune visite de monitoring ne sera réalisée dans les sites des Médecins Observateurs par les moniteurs du Laboratoire Aventis (ou par des représentants). La cohérence et la qualité des données contenues dans les fiches d'observation complétées par les Médecins Observateurs seront contrôlées par la société prestataire de services en charge de la gestion des données de l'étude **(MédiSCAN – I.B.S)** à réception de ces fiches. Lorsque nécessaire, des demandes de corrections des données des fiches d'observations ou des demandes de compléments d'information seront faites par la société de services en charge de la gestion des données de l'étude auprès des Médecins Observateurs.

Des audits sur site pourront être réalisés par les auditeurs du Laboratoire Aventis.
Le nombre, le tirage au sort et les dates d'audit des Médecins Observateurs seront fonction du plan de qualité relatif aux études en cours défini par le département Assurance de Qualité Clinique.
Les conditions de ces audits seraient :

- Nombre d'audit compris entre 3 et 5 % des Médecins Observateurs actifs (ayant inclus au moins un patient)
- Sélection de ces Médecins Observateurs par tirage au sort à partir de la liste des Médecins Observateurs actifs lorsque 50 à 60% des Médecins Observateurs participant auront inclus.
- Les données à contrôler seront définies lors du plan d'audit réalisé par le département Assurance de Qualité Clinique du Laboratoire Aventis.

8.9 Archivage

Les documents de l'étude doivent être archivés par le Médecin Observateur jusqu'à ce que la destruction de ces documents soit autorisée par le Laboratoire Aventis.

8.10 Responsabilité et assurance du promoteur

Cette étude observationnelle n'interfère pas avec l'exercice normal de la médecine, et rentre dans le cadre de l'assurance des praticiens pour "responsabilité civile professionnelle". En outre, les produits ou dispositifs utilisés pour le suivi ou le soin des sujets ne sont pas prescrits pour les besoins de cette étude et font l'objet d'une assurance responsabilité civile professionnelle des sociétés commercialisant ces produits ou dispositifs.

9. DOCUMENTATION ET UTILISATION DES RESULTATS DE L'ETUDE

9.1 Documentation de l'étude

Le promoteur fournira à chaque Médecin Observateur:

- 1 Protocole.
- 1 Fiche d'engagement du Médecin Observateur.
- 2 Conventions financières.
- L'avis du CNOM.
- Une lettre du Comité de Pilotage.
- 4 Formulaires d'information sujet
- 1 Guide d'instructions pour compléter les fiches de recueil d'information.
- 1 Fiche de recueil d'information Médecin.
- 4 Fiches de recueil d'information sujet V1.
- 4 Fiches de recueil d'information sujet V2.

Pour les Médecins Observateurs faisant partis des régions du Groupe "Guide du dos" il leur sera fourni 5 exemplaires du Guide du dos: 1 exemplaire à remettre à chacun des 4 patients inclus et un exemplaire pour chacun d'eux afin de s'en informer.

Toutes les données recueillies dans le cadre du protocole devront être transcrites dans la fiche d'observation par le Médecin Observateur. Les modalités de remplissage et de corrections de ces fiches seront expliquées au Médecin Observateur.

Le Médecin Observateur devra remplir la fiche d'observation le plus tôt possible après le recueil de l'information, de préférence le jour même de la consultation, de la visite ou de l'appel téléphonique.

Toutes les données en suspens devront être transcrites le plus rapidement possible après la visite finale. Il faudra fournir une explication pour toutes les données manquantes.
Une fois remplie, la fiche d'observation devra être revue par le Médecin Observateur signataire du protocole.
Le Médecin Observateur conservera un original sous forme "duplicate" des fiches d'observation.
Les originaux de toutes les fiches de recueil des données seront conservés par le promoteur.

9.2 Utilisation des résultats de l'étude

Toutes les informations relatives à cette étude et non encore publiées, sont confidentielles et demeurent la seule propriété du promoteur. Le Médecin Observateur s'engage à n'utiliser ces informations que pour la conduite de l'étude et pour aucun autre motif sauf accord préalable écrit du promoteur.

En signant le protocole, le Médecin Observateur accepte que les résultats de l'étude soient utilisés à des fins de publication et information des professionnels médicaux et pharmaceutiques. Si nécessaire, les nom, adresse, qualifications et rôle dans l'étude du Médecin Observateur seront publiés.

Le promoteur rédigera un rapport final de l'étude.

Tous les documents fournis par le promoteur au Médecin Observateur ainsi que les fiches d'observation complétées par le Médecin Observateur et relatives à l'étude sont la seule et unique propriété du promoteur.

Les modalités selon lesquelles les publications seront écrites, seront définies avant le début de l'étude d'un commun accord entre d'une part le Comité Scientifique et d'autre part le promoteur.

10. ENGAGEMENTS DU PROMOTEUR ET DU MEDECIN OBSERVATEUR

10.1 Engagement du promoteur

Le protocole a été revu et approuvé par le comité de lecture du promoteur ainsi que le comité scientifique de l'étude. L'information contenue est conforme à la réglementation française.
Le Médecin Observateur sera informé de toutes les données nouvelles ou significatives ainsi que de tout élément nouveau concernant l'étude.

Responsable du projet

Date:_________ Signature : ___________________________________

Nom: Fernand CORIAT

Statisticien

Date:_________ Signature : ___________________________________

Nom: Sylvie BRIN

10.2 Engagement du Médecin Observateur

J'ai lu le protocole et confirme qu'il contient toute l'information nécessaire à la conduite de l'étude. Je m'engage à réaliser l'étude telle que décrite dans le protocole.

Je n'inclurai pas de sujet dans l'étude avant que ce protocole n'ait obtenu l'accord des autorités compétentes et avant d'avoir personnellement satisfait à toutes les exigences légales françaises et avant que le Conseil de l'Ordre National des Médecins n'ait donné son accord quant à la rémunération proposée

Je conduirai l'étude en accord avec la réglementation française.

Je m'engage à informer chaque sujet de la nature, de l'objectif et des conséquences possibles de l'étude ainsi que de l'existence d'un traitement informatisé des données nominatives, des finalités de ce recueil et de son droit d'accès et de rectification. Je m'engage à remettre à chaque sujet le formulaire d'information préparé pour cette étude par le Laboratoire Aventis.

Je donne mon accord quant à l'utilisation des résultats de cette étude dans un dossier d'enregistrement national, dans une publication, dans un document d'information destiné aux professionnels médicaux et pharmaceutiques.

Je m'engage à conserver toute la documentation liée à l'étude comme indiqué au chapitre *Archivage* du présent protocole.

Le Médecin Observateur

Date:_________ Signature : _______________________________

Nom (en lettres capitales) : ___________________

11. REFERENCES

1. Prise en charge diagnostique et thérapeutique des lombalgies et des lombosciatiques communes de moins de trois mois d'évolution.
   ANAES. Texte des recommandations, février 2000.
2. Leboeuf-Yde C, Klougart N, Lauritzen T. How common is low back pain in the Nordic population? Data from a recent study on a middle-aged general Danish population and four surveys previously conducted in the Nordic countries.
   Spine 1996;21:1518-26.
3. Papageorgiou AC, Croft PR, Ferry S, Jayson MIV, Silman AJ. Estimating the prevalence of low back pain in the general population. Evidence from the South Manchester Back Pain Survey.
   Spine 1995;20:1889-94.
4. Haut Comité de la santé publique. Le mal de dos. La santé en France. Rapport général.Paris:
   Documentation Française; 1998. p.275-7.
5. Valat JP et coll. Indice prédictif de l'évolution chronique des lombalgies aiguës. Elaboration par l'étude d'une cohorte de 2487 patients.
   Rev.rhum. (Ed. Fr), 2000, 67; 528-35.
6. Valat JP. Epidémiologie des lombalgies.
   Rev.rhum. (Ed. Fr), 1998, 65 (5 bis), 1725-1745.
7. Diagnostic, prise en charge et suivi des malades atteints de lombalgie chronique.
   ANAES. Texte des recommandations, décembre 2000.
8. Waddell G, Newton M, Henderson I, Somerville D, Main CJ Fear-Avoidance Beliefs Questionnaire (FABQ) and the role of fear-avoidance beliefs in chronic low back pain and disability.
   Pain 1993 Feb; 52 (2): 157-68.
9. Duquesnoy B, Allabert FA, Verdoncq B. Retentissement psycho-social et professionnel de la lombalgie chronique.
   Rev. rhum (Ed. Fr), 1998, 65 (1), 37-45.
   Rev.rhum (Engl. Ed), 1998, 65 (1), 33-40
10. Chaory K, Poiraudeau S, Lefebvre-Colau MM, Fermanian J, Revel M. Validation d'une version française du Fear-Avoidance Belief Questionnaire (FABQ).
    Congrès de la Société Française de Rhumatologie, Novembre 2002, Paris.
11. Information des patients. Recommnadations destinés aux médecins.
    ANAES, services des recommandations et références professionnelles, 1999, Paris, France.
12. Burton AK, Waddel G, Tillotson KM, Summerton N. Information and advice to patients with back pain can have a positive effect. A randomized controlled trial of a novel educational booklet in primary care.
    Spine 1999 ; 24 : 2484-91.
13. Buchbinder R, Jolley D, Wyatt M. Population based intervention to change back pain beliefs and disability : three part evaluation.
    BMJ 2001 ; 322 : 1516-20.
14. Bouyer J, Hémon D, Cordier S, Derriennic F, Stücker I, Stengel B, Clavel J. Epidémiologie: Principes et méthodes quantitatives.
    Les Editions INSERM 1993; Chapitre 21: 424-438.
15. Coudeyre E, Givron P, Gremeaux V, Lavit P, Hérisson C, Combe B, Burton AK, Poiraudeau S. Le "Guide du Dos", traduction française et adaptation culturelle du "Back Book".
    Ann Réadaptation Med Phys 2003 (Article accepté et sous presse).

16 Bougault.A et coll. Relais thérapeutiques dans le traitement des douleurs postopératoires. Réunion des groupes de travail Spécifiques – Congrés SFD-SOFRED 2000.

17 Garrec. F et coll. Evaluation de la douleur aiguë à l'urgence et en pré-hospitalier. Elsevier. Paris 1999, Actualités en réanimation et urgence.

18 Kopec JA, and all. The Quebec Back Pain Disability Scale. Measurement properties. Spine. 1995 Feb 1; 20(3): 341-52

ANNEXES

- Annexe 1: Sectorisation de la France en 30 Régions selon le découpage régional de la Visite Médicale.

- Annexe 2: Régions groupe "Témoin" et régions groupe "Guide du dos".

- Annexe 3: Formulaire d'information des sujets.

Annexe 1:

1.1. Sectorisation de la France en 30 Régions selon le découpage régional du Laboratoire Aventis

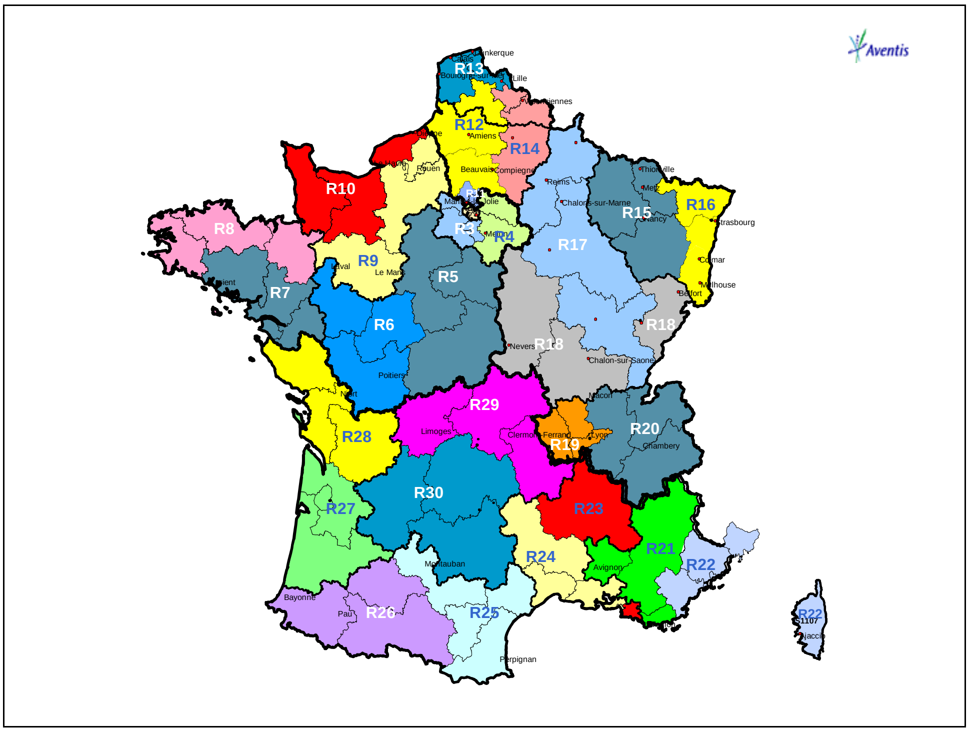

1.2. PARIS et Région Parisienne

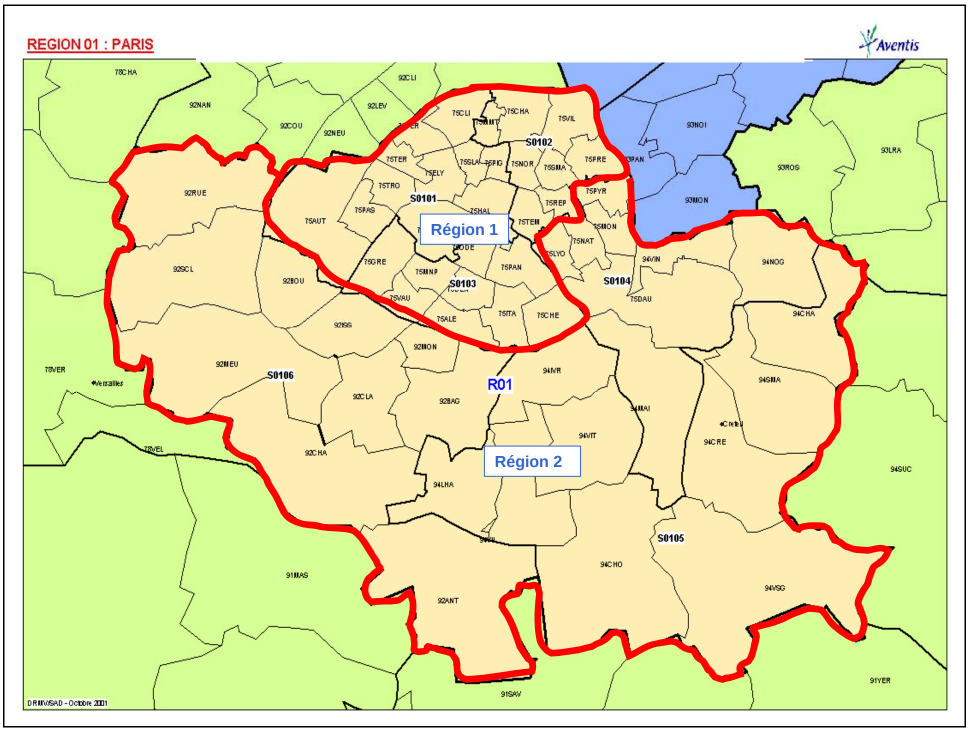

Annexe 2:

Régions groupe "Témoin" et régions groupe "Guide du dos".

Régions Groupe Témoin
1
3
5
7
9
11
13
15
17
19
21
23
25
27
29

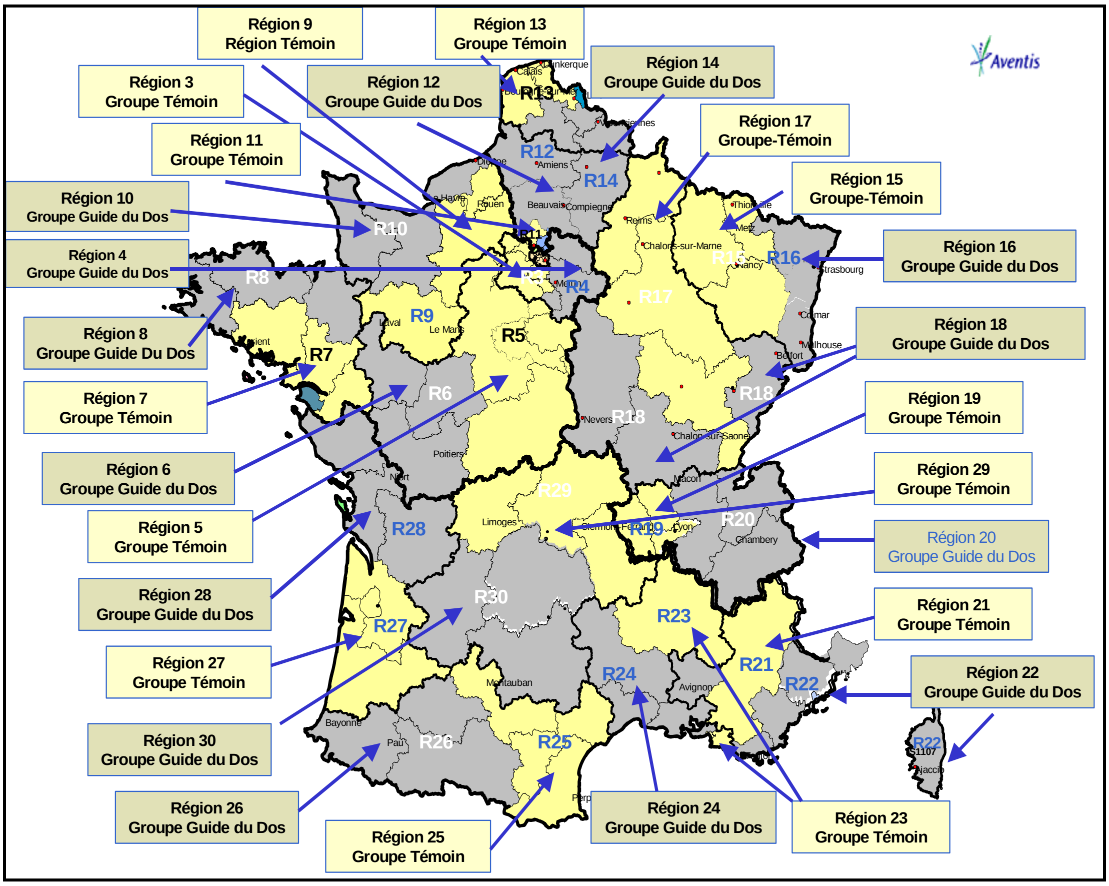

Régions Groupe Guide du Dos
2
4
6
8
10
12
14
16
18
20
22
24
25
28
30

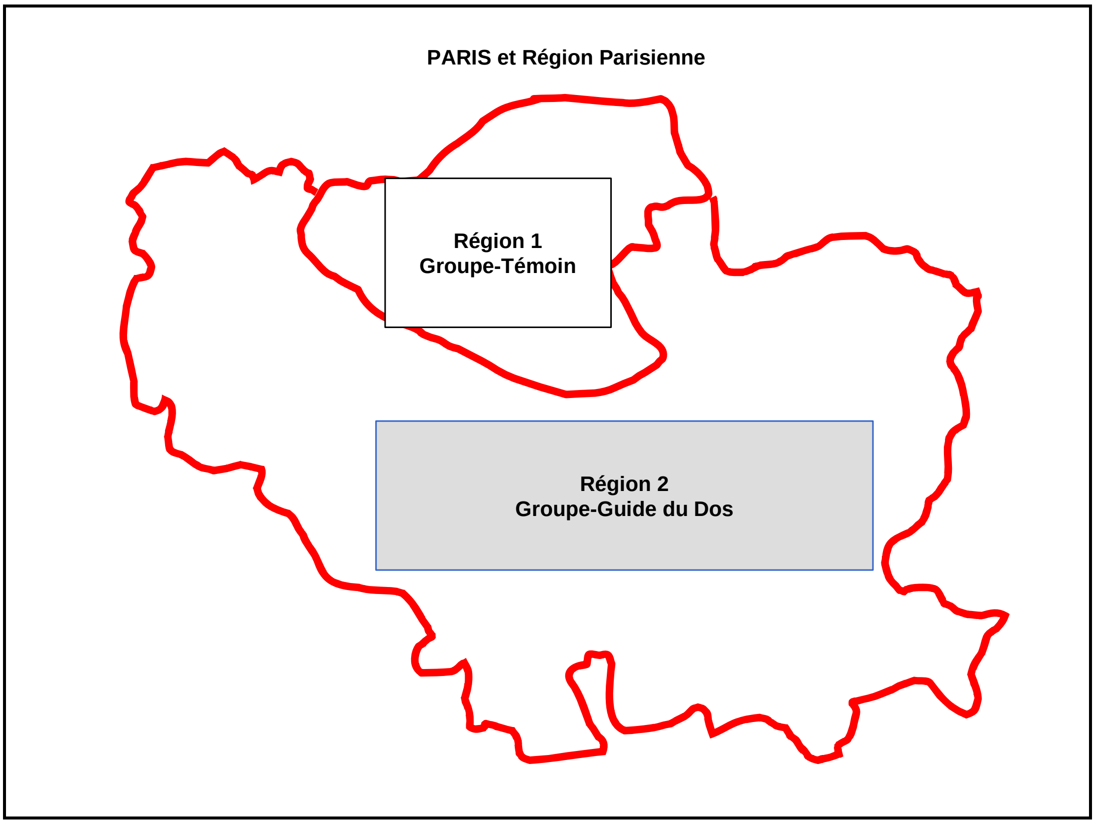

Annexe 3:

Formulaire d'information des sujets.

Madame, Monsieur,

Nous vous remercions d'avoir accepté de participer à cette étude qui concerne la lombalgie commune dont vous souffrez. Son but est d'évaluer l'impact d'un programme éducatif par l'information orale et/ou écrite "Le Guide du dos" sur l'évolution de votre lombalgie.
Cette étude se réalise en 2 temps, aujourd'hui et dans environ 3 mois.

Cela nous conduit à vous interroger aujourd'hui et dans environ 3 mois sur :

Vous, votre maladie et l'impact de votre maladie sur vos activités quotidiennes

Qu'attendons-nous de vous aujourd'hui ?

Que vous répondiez en toute franchise aux différentes questions que vous posera votre médecin sur votre mal de dos.
Dans environ trois mois, si cela est nécessaire, vous retournerez voir votre médecin ou il vous contactera par téléphone pour savoir comment vous allez. Pour cela, il vous posera des questions similaires à celles auxquelles vous acceptez de répondre aujourd'hui.

Afin de vous rassurer sur notre démarche, sachez que tous les renseignements recueillis resteront totalement anonymes, pour les personnels de santé concernés, les laboratoires, les médecins, les pharmaciens. Tous sont garants de cette confidentialité.

Le fichier informatique utilisé pour réaliser cette étude a fait l'objet d'une autorisation de la CNIL en application des articles 40-1 et suivants de la loi "Informatique & Libertés". Le recueil d'informations vous concernant nécessaire à cette étude fera l'objet d'un traitement informatique et ne sera transmis qu'au Laboratoire Aventis ou à son représentant. Vous disposez d'un droit d'accès et de rectification et d'opposition relatif à votre dossier médical, que vous pourrez exercer en vous adressant soit directement à votre médecin traitant, soit à un médecin de votre choix qui sera alors votre intermédiaire.

Vous pourrez obtenir auprès de votre médecin qui vous a proposé de participer à l'étude toute information complémentaire en cours d'étude et être informé des résultats globaux de l'étude à la fin de celle-ci, comme le prévoit l'article 15 de la loi numéro 2002-303 du 04 mars 2002.

Vous pouvez donc répondre sans inquiétude aux questions qui vous sont proposées.

Nous vous remercions par avance de votre participation et nous vous prions de croire à nos salutations les meilleures.
