# Supplementary material for: Effect of a Simple Information Booklet on Pain Persistence after an Acute Episode of Low Back Pain: A Non-Randomized Trial in a Primary Care Setting
Source: PLoS One. 2007 Aug 8;2(8):e706. doi: 10.1371/journal.pone.0000706 (PMC1939729; doi:10.1371/journal.pone.0000706)
Supplement: Alternate Language Abstract S1 — Translation of the abstract into French by Serge Poiraudeau (0.02 MB DOC) [file pone.0000706.s003.doc]

**Résumé**

**Objectif:** Des campagnes médiatiques peuvent modifier l’évolution des lombalgies. Nous avons évalué l’impact sur l’évolution de lombalgies aiguës d’un livret d’informations standardisées.

Methodes:

*Type d’étude:* Etude pragmatique multicentrique contrôlée avec stratification géographique, d’une durée de 3 mois.

*Contexte:* Soins primaires en France.

*Participants:* 2752 patients souffrant de lombalgies aiguës.

*Intervention:* Un livret d’information sur la lombalgie (le “guide du dos”).

*Critère d’efficacité:* Le critère principal d’efficacité était la persistance d’une douleur lombaire à 3 mois.

**Résultats:** 2337 (85%) patients ont été évalués à 3 mois et 12.4% d’entre eux rapportaient des lombalgies persistantes. La réduction du risque absolu de rapporter des douleurs persistantes dans le groupe intervention était de **3.6% par rapport au groupe contrôle (10.5% vs 14.1%; Intervalle de confiance à 95% [-6.3% ; -1.0%]; valeur de p ajustée pour l’effet cluster = 0.01)**. Les patients dans le groupe contrôle étaient plus satisfaits que ceux du groupe contrôle pour l’information reçue sur les activités physiques, quand consulter son médecin, et comment prévenir un nouvel épisode douloureux. Le nombre de patients ayant eu un arrêt de travail et la durée de ceux-ci ne différaient pas dans les 2 groupes. Parmi les patients rapportant des douleurs persistantes, il n’y avaient pas d’effet du « guide du dos » sur les incapacités et les peurs et croyances.

**Conclusions**: Les effets de ce livret sont modestes mais le coût et la complexité de l’intervention sont minimes. La généralisation de cette d’intervention dans ce contexte est souhaitable.
